# Widening the Prostacyclin Paradigm: Tissue Fibroblasts Are a Critical Site of Production and Antithrombotic Protection

**DOI:** 10.1161/ATVBAHA.123.318923

**Published:** 2023-10-12

**Authors:** Maria Vinokurova, Maria Elisa Lopes-Pires, Neringa Cypaite, Fisnik Shala, Paul C. Armstrong, Blerina Ahmetaj-Shala, Youssef Elghazouli, Rolf Nüsing, Bin Liu, Yingbi Zhou, Chuan-ming Hao, Harvey R. Herschman, Jane A. Mitchell, Nicholas S. Kirkby

**Affiliations:** National Heart and Lung Institute, Imperial College London, United Kingdom (M.V., M.E.L.-P., N.C., F.S., B.A.-S., Y.E., J.A.M., N.S.K.).; Blizard Institute, Queen Mary University of London, United Kingdom (P.C.A.).; Clinical Pharmacology and Pharmacotherapy Department, Goethe University, Frankfurt, Germany (R.N.).; Cardiovascular Research Centre, Shantou University Medical College, China (B.L., Y.Z.).; Division of Nephrology, Huashan Hospital, Fudan University, Shanghai, China (C.-m.H.).; Department of Molecular and Medical Pharmacology, University of California Los Angeles (H.R.H.).

**Keywords:** endothelial cells, fibroblasts, immunoassay, risk factors, thrombosis

## Abstract

**BACKGROUND:**

Prostacyclin is a fundamental signaling pathway traditionally associated with the cardiovascular system and protection against thrombosis but which also has regulatory functions in fibrosis, proliferation, and immunity. Prevailing dogma states that prostacyclin is principally derived from vascular endothelium, although it is known that other cells can also synthesize it. However, the role of nonendothelial sources in prostacyclin production has not been systematically evaluated resulting in an underappreciation of their importance relative to better characterized endothelial sources.

**METHODS:**

To address this, we have used novel endothelial cell–specific and fibroblast-specific COX (cyclo-oxygenase) and prostacyclin synthase knockout mice and cells freshly isolated from mouse and human lung tissue. We have assessed prostacyclin release by immunoassay and thrombosis in vivo using an FeCl_3_-induced carotid artery injury model.

**RESULTS:**

We found that in arteries, endothelial cells are the main source of prostacyclin but that in the lung, and other tissues, prostacyclin production occurs largely independently of endothelial and vascular smooth muscle cells. Instead, in mouse and human lung, prostacyclin production was strongly associated with fibroblasts. By comparison, microvascular endothelial cells from the lung showed weak prostacyclin synthetic capacity compared with those isolated from large arteries. Prostacyclin derived from fibroblasts and other nonendothelial sources was seen to contribute to antithrombotic protection.

**CONCLUSIONS:**

These observations define a new paradigm in prostacyclin biology in which fibroblast/nonendothelial-derived prostacyclin works in parallel with endothelium-derived prostanoids to control thrombotic risk and potentially a broad range of other biology. Although generation of prostacyclin by fibroblasts has been shown previously, the scale and systemic activity was unappreciated. As such, this represents a basic change in our understanding and may provide new insight into how diseases of the lung result in cardiovascular risk.

**Highlights**

- Prostacyclin—a powerful antithrombotic mediator—is abundantly produced by both arteries and in tissues, especially the lung.
- In arteries, prostacyclin production is produced by vascular endothelial cells, but in the lung and other tissues, prostacyclin production is essentially independent of the endothelium.
- Lung microvascular endothelial cells are weak producers of prostacyclin by comparison to arterial endothelial cells.
- As such, prostacyclin production in the lung is associated with fibroblasts (both vascular and nonvascular) and contributes to systematic antithrombotic protection.
- This previously unappreciated degree to which nonendothelial sources in the lung and elsewhere contribute to prostacyclin production represents a new paradigm in prostacyclin biology and may explain the associations between lung disease and thrombotic risk.

**See accompanying editorial on page 287**

Prostacyclin is a powerful endogenous inhibitor of platelet activation and represents one of the bodies of fundamental antithrombotic and cardioprotective pathways.^1,2^ Inhibition/deficiency of the I-prostanoid receptor (IP) through which prostacyclin acts results in a prothrombotic phenotype in animal models^3,4^ and an increased risk of heart attacks and strokes in man.^5^ Prostacyclin can also, in some contexts, act as a vasodilator^1^ and protect against atherogenesis.^6^ Dysfunction of the prostacyclin pathway is a feature of vascular pathologies, including pulmonary hypertension where exogenous prostacyclin is an established therapy.^2^ In addition to its cardiovascular actions, prostacyclin is important in lung, gastrointestinal and renal function, in pain/inflammation, and the regulation immunity. As such, understanding prostacyclin biology is essential not only for cardiovascular health but also for the proper functioning of a broad range of organ systems.

Prostacyclin is produced as part of a family of prostanoid mediators by the activities of phospholipases, COXs (cyclo-oxygenases), and prostacyclin synthase, through stepwise metabolism of membrane lipids.^2^ Phospholipases, principally the cytosolic phospholipase A_2_ isoform,^7^ liberate the substrate, arachidonic acid, from cell membranes, which is then converted to an unstable intermediate, prostaglandin H_2_ (PG H_2_), by 2 isoforms of the COX enzyme. COX-1 is, like cytosolic phospholipase A_2_, widely expressed as a physiological housekeeping enzyme. In contrast, constitutive cyclo-oxyenase-2 expression is restricted to certain regions including the kidney, brain, and gut^8^ but can be induced elsewhere during inflammation and proliferation. Both COX isoforms contribute to prostacyclin generation and cardiovascular protection through distinct but parallel pathways. COX-2 drives prostacyclin metabolites in the urine^9^ (the origin of which is controversial^10^) but where studied directly, does not appear to mediate prostacyclin production by isolated systemic mouse^11,12^ or human arteries^13^ except under conditions of gross inflammation.^14^ It has been suggested that vascular COX-2 is rapidly lost when vessels are taken away from the influence of blood flow since laminar shear increases COX-2 in endothelial cells.^15^ However, while acute application of shear to static endothelial cultures has been shown to increase COX-2 expression,^15^ this is not seen in endothelial cells subject to chronic laminar shear stress.^16^ Nonetheless, COX-2 plays an unequivocal role in cardiovascular health; its inhibition in man increases cardiovascular risk,^17^ and vascular COX-2 deletion in mouse models increases thrombosis.^4,18^ COX-1 has a more clearly defined role in bulk systemic prostacyclin generation; COX-1 deletion/inhibition abolishes prostacyclin production by isolated mouse^11,12^ and human vessels,^13^ and consequently, vascular COX-1 deficiency is associated with acceleration of the thrombosis.^4^

The final step in prostacyclin synthesis is conversion of PG H_2_ to prostacyclin by the enzyme prostacyclin synthase, which has a narrower expression pattern, resulting in spatial differences in prostacyclin production. Prostacyclin was first discovered from arterial endothelial cells,^4,19^ and since then, arterial tissue ex vivo and arterial endothelial cells in vitro have been almost universally observed to possess a robust capacity for prostacyclin synthesis. By comparison, platelets^1^ and leucocytes^20^ are almost entirely deficient in their ability to physiologically produce prostacyclin. It was quickly realized that prostacyclin was also produced by isolated tissues; it is abundant in tissue perfusates and the major prostanoid generated by the lung and the heart.^21,22^ In vitro evidence has indicated many cell types, including epithelial cells,^23^ fibroblasts,^24–26^ and smooth muscle cells (SMCs),^27^ have at least some prostacyclin synthetic capacity, but it remains unknown within intact tissues whether these sources contribute meaningfully to prostacyclin production or whether this is instead associated with their vascular compartments. This question has remained unanswered because there have been no tools available that allow the contribution of individual cell types to be assessed for prostacyclin production in intact tissues or in vivo. We have recently described mice in which COX-1, COX-2,^4^ or prostacyclin synthase^28^ can be deleted specifically from vascular endothelial cells, which provide new tools to identify the cellular origins of prostacyclin within tissues. In the current study, we have used them to determine whether prostacyclin synthesis is purely a product of the endothelium or whether there are additional, underappreciated depots of prostacyclin production in the body that contribute to local organ function or systemic antithrombotic protection.

## MATERIALS AND METHODS

The authors declare that all supporting data are available within the article (and its Supplemental Material). Supplemental Material and resources are available from the corresponding author upon reasonable request.

### Animal Studies

Studies were performed on 8- to 12-week-old male and female mice housed in individually ventilated cages with free access to standard laboratory diet and water and 12-hour day/night cycle. All procedures were conducted in accordance with the UK Animals (Scientific Procedures) Act (1986) Amendment (2013) and the Guide for the Care and Use of Laboratory Animals published by the US National Institutes of Health (publication number 85-23, revised 1996) and after local approval from the Imperial College Animal Welfare Ethical Review Board (UK Home Office License Project Licenses 70/7013 and PP1576048) or Fudan University. Unless otherwise indicated, animals were euthanized by CO_2_ narcosis. Tissue samples were collected and used to measure prostaglandin content, prostaglandin release, and gene expression,^4,8,12,29,30^ and thrombotic responses in vivo were measured using the FeCl_3_ carotid artery injury model^4,29^ according to our previously published methodology.

Global COX-1 knockout mice (*Ptgs1*^*−/−*^)^31^ were generated as described previously and compared with age- and sex-matched wild-type C57Bl/6J controls (Charles River, United Kingdom). Endothelial-specific COX-1 knockout mice (*Ptgs1*^*flox/flox*^*; VEC-iCre*) were generated as described previously ^4^ using VEC (VE-cadherin) Cre^ERT2^, which is thought to be highly selective for endothelial cells with no off-target expression reported.^32^ Because this Cre requires activation by tamoxifen, at 4 to 6 weeks of age, all mice from this line were treated with tamoxifen (50 mg/kg, ip, QD for 5 days; Sigma, United Kingdom) and allowed to recover to 2 weeks before further use. Smooth muscle–specific COX knockout mice (*Ptgs1*^*flox/flox*^*; Tagln-Cre*)^33^ were generated as described previously^4^ using *Tagln*-Cre, which is thought to have some potential off-target expression in cardiomyocytes,^4^ platelets, adipocytes, and myeloid cells.^34^ Endothelial/platelet-specific COX-1 knockout mice (*Ptgs1*^*flox/flox*^*; Tek-Cre*),^4^ endothelial/platelet-specific COX-2 knockout mice (*Ptgs2*^*flox/flox*^*; Tek-Cre*),^4^ and endothelial/platelet-specific prostacyclin synthase (*Ptgis*^*flox/flox*^*; Tek-Cre*)^28^ were generated using *Tek*-Cre, which is thought to have some potential off-target expression in heart valves and hematopoietic cells.^32^ Fibroblast-specific COX-1 knockout mice (*Ptgs1*^*flox/flox*^*; Fsp1* [fibroblast-specific protein-1]*-Cre*) were generated by crossing Floxed *Ptgs1* mice^4^ with transgenic mice harboring *Fsp1/S1004A4-Cre*,^35^ which is thought to have some potential off-target expression in macrophages.^36^ These strains were validated for effective deletion of the Floxed gene in target cells and retention in select nontarget cells either by our previous work^4,28^ or in the current study, but we cannot exclude expression in all nontarget cells including those highlighted above, which can be considered a limitation of these models. These validation data are summarized in Figure S1. Mouse models were maintained on a pure C57Bl/6J background (*Ptgis* models); a mixed C57Bl/6J, 129S4/SvJae, and BALB/c background (*Ptgs1*^*flox/flox*^*; Fsp1-Cre* model); or a mixed C57Bl/6J and 129S4/SvJae background (other *Ptgs1*/*Ptgs2* models). EGFP (enhanced green fluorescent protein)/Cre activity reporter strains were generated by crossing ROSA^mT/mG^ mice (Jackson Laboratories strain: 7676)^37^ with transgenic *Tek*-Cre^38^ or Fsp1/*S100A4*-Cre mice and were studied as heterozygous animals. For all cell-specific knockout strains, animals were genotyped by genomic polymerase chain reaction to identify Cre-positive knockout animals from the Cre-negative littermates, which were used as experimental controls for each strain. Genotyping was performed at weaning only from ear clip material by Charles River Laboratories, United Kingdom, in a blind manner. Further details of animal strains and ARRIVE (Animals Research: Reporting of In Vivo Experiments) reporting are given in the Major Resources Table.

### Human Tissue Studies

All studies using human material were conducted in accordance with the Declaration of Helsinki, and samples were donated from volunteers and patients who gave explicit informed consent. Blood was collected into sodium citrate (0.32% final; BD Biosciences, Germany) from healthy male and female volunteers aged 18 to 40 years after ethical approval by the West London & GTAC Research Ethics Committee (approval 15/LO/223) or the St Thomas’ Hospital Research Ethics Committee (approval 07/Q0702/24). Lung parenchyma and pulmonary artery were collected from patients undergoing surgical lung resection for treatment adenocarcinoma, small cell carcinoma, or squamous carcinoma. Histologically normal areas of the resected tissue were identified for study by a clinical pathologist. Five male and 5 female patients with an average age of 70.2 (range, 54–84) years donated lung parenchyma samples. Of these, 3 male and 2 female patients with an average age of 67.6 (range, 57–78) years also donated pulmonary artery. Lung tissue was provided through the Royal Brompton and Harefield NHS Trust Biomedical Research Unit Advanced Lung Disease Biobank after ethical approval by the South Central–Hampshire B Research Ethics Committee (approval 15/SC/0101) and local project review by the Biomedical Research Unit Heads of Consortia (approval JM04).

### Tissue and Plasma Prostanoid Levels

#### Tissue/Vessel Segments

Tissue segments (≈10 mm^3^) or vascular rings (≈2 mm long) were cleaned of adherent material, transferred to 96-well microplate wells containing A23187 (Ca^2+^ ionophore, 30 μM; Sigma) or arachidonic acid (30 μM; Sigma) in DMEM media (Sigma). After 30 minutes of incubation at 37 °C, the supernatant was collected. Where indicated, before tissue collection, the lungs were flushed of blood by perfusion of the pulmonary vasculature with PBS via the right ventricle with effective perfusion confirmed by blanching of the lung tissue. In some experiments, tissues were preincubated with the selective COX-1 (SC-560, 1 µM; Abcam Laboratories, United Kingdom) or COX-2 (rofecoxib, 1 µM; Sigma) for 90 minutes before stimulation. In some experiments, aortic rings were denuded of endothelial cells by rubbing of the luminal surface with fine forceps, then snap-frozen for RNA extraction.

#### Blood

Blood was collected from the inferior vena cava into heparin (10 U/mL final; Leo Laboratories, United Kingdom) and stimulated with A23187 (30 μM) for 30 minutes, before centrifugation (8000*g*, 2 minutes) and separation of conditioned plasma.

#### Homogenates

Segments of lung parenchyma were removed and snap-frozen. Lung segments were suspended in 10× volume of ice-cold PBS containing complete mini protease inhibitor cocktail (Roche, Switzerland), 2 mM EDTA (Sigma), and an excess of the nonselective COX inhibitor, diclofenac (1 mM; Sigma). Samples were immediately homogenized using a Precellys24 instrument (Bertin Instruments, France) and the supernatant collected after centrifugation (8000*g*, 2 minutes).

#### Plasma

Blood was collected from the inferior vena cava into heparin (10 U/mL final) immediately postmortem. Plasma was separated by centrifugation (8000*g*, 2 minutes) and stored.

#### Perfusates

Immediately postmortem, the thoracic cavity was opened and the pulmonary vasculature flushed of blood with PBS via the right ventricle. The right atrium of the heart was then cannulated with PE10 tubing, secured with 5-0 silk, and the lung and heart were removed intact. The pulmonary vasculature was perfused via the right atrium with DMEM media at 37 °C for 20 minutes using a peristaltic pump at 50 μL/min and the venous outflow collected from the left atrium.

Levels of the prostacyclin breakdown product 6-keto PG F1_α_ (6-ketoPGF_1α_), the thromboxane breakdown product thromboxane B_2_, prostaglandin E_2_, prostaglandin F_2α_, prostaglandin D2, 12-HETE, and 15-HETE (Cayman Chemical) were measured in supernatants/plasma by commercial immunoassay. In some cases, tissue/vessel segments were weighed and prostanoid levels expressed relative to tissue mass.

### RT-qPCR

Tissue segments were snap-frozen, then homogenized in ice-cold RLT buffer (Qiagen, United Kingdom) containing β-mercaptoethanol (1% v/v; Sigma) using a Precellys24 instrument. RNA was extracted using RNeasy mini-prep kits (Qiagen) and gene expression levels measured using a 1-step RT-qPCR (reverse transcriptase quantitative polymerase chain reaction) master mix (Promega, United Kingdom) and a 7500 Fast qPCR instrument (Applied Biosystems, United Kingdom) using TaqMan probes (Qiagen) recognizing *Ptgis* (probe ID: Mm00447271_m1), *Ptgs1* (probe ID: Mm00477214_m1), *Ptgs2* (probe ID: Mm00478374_m1), or the housekeeping genes *18S* (probe ID: Mm03928990_g1) and *Gapdh* (probe ID: Mm99999915_g1). Data were analyzed by the comparative threshold method, with relative expression levels normalized to those to 18S and *Gapdh* and experimental control groups.

### Platelet Prostacyclin Bioassay

A human platelet bioassay was used to measure bioactive prostacyclin levels released from segments of mouse lung parenchyma (≈10 mm^3^). Platelet-rich plasma (PRP) and platelet-poor plasma were separated from human blood by centrifugation (PRP: 230*g*, 15 minutes; platelet-poor plasma: 8000*g*, 2 minutes). PRP was preincubated with aspirin (30 μM, 30 minutes prior; Sigma) and diethylamine NONOate (10 μM, 1 minute prior; Sigma) to sensitize platelets to prostacyclin. Lung segments were added to individual wells of 96-well microtiter plates containing PRP and preincubated for 1 minute, before stimulation of platelets and tissues with A23187 (30 μM) and vigorous mixing (1200 revolutions per minute, BioshakeIQ; Q Instruments, Germany). After 5 minutes, the tissue segments were removed and the absorbance of each well at 595 nm measured by spectrophotometer and the amount of platelet aggregation calculated by reference to the absorbance of unstimulated PRP (0% aggregation) and platelet-poor plasma (100% aggregation).

### Mouse Lung Fibroblast Culture

For validation of fibroblast COX-1 knockout mice, lung fibroblast cultures were established using the explant method. Finely minced lung tissue was partially digested with collagenase I (5 mg/mL; Sigma) in PBS, then cultured in DMEM media for 10 days with regular media changes to remove debris until a homogenous monolayer of fibroblasts grew out. Cells were lysed in the RLT buffer (Qiagen) containing β-mercaptoethanol (1% v/v; Sigma) for RNA extraction.

### Human Lung Cell Culture

Human primary lung microvascular endothelial cells (1 female and 2 male donors; Lonza, Germany) and human primary lung fibroblasts (3 female donors; Promocell, Germany) from 3 individual donors each were cultured according to suppliers’ instructions in full endothelial growth factor-2 media (Promocell) supplemented with 10% fetal calf serum (Biosera, United Kingdom) and penicillin/streptomycin (Sigma). At passages 4 to 8, cells were plated in 96-well culture plates at a density of 10 000 cells per well in the same media and allowed to settle overnight. The following day, media was replaced and cells stimulated with arachidonic acid (30 μM) for 30 minutes at 37 °C before collection of media for measurement of the stable prostacyclin breakdown product 6-ketoPGF_1α_ by immunoassay. Cells were fixed and counted to confirm the density remained the same between types at the point of stimulation.

### Thrombosis

Under isoflurane anesthesia, the left carotid artery was exposed and separated from the attached nerve and vein. FeCl_3_ (4%–6%; Sigma) was applied to the adventitial surface of the vessel for 3 minutes, then the vessel irrigated, and a Doppler perivascular flow probe (Transonic Systems, United Kingdom) was secured around the artery. For each batch of experiments, the FeCl_3_ concentration applied was titrated to achieve a threshold injury in the control group (≈25% rate of thrombotic occlusion) to provide the maximum window to observe a prothrombotic modulation of the treatment/genotype. Blood flow was recorded for 30 minutes and time to occlusion recorded as the time taken from injury to the first point at which blood flow dropped to <10% of baseline. If no occlusion occurred, occlusion time was recorded as 30 minutes. In some experiments, after anesthesia, the COX-1 inhibitor SC-560 (10 mg/kg; Cayman Chemical) or vehicle (5% dimethyl sulphoxide) were administrated intravenously (tail vein) 15 minutes before arterial injury.

### Fluorescence-Activated Cell Sorting

Mouse lung, mouse aorta, human lung parenchyma, and human pulmonary artery were finely minced with scissors in an enzyme cocktail of collagenase I (5 mg/mL; Sigma), DNase I (125 U/mL; Sigma), and elastase (100 μg/mL; Sigma) in PBS containing CaCl_2_ (2 mM; Sigma) and incubated at 37 °C with regular mixing until fully digested. Cycloheximide (3 μM; Sigma) was added to this and all solutions to prevent artifactual changes in prostanoid pathways during cell isolation. Erythrocytes were lysed using ammonium-chloride-potassium lysis buffer (Life, United Kingdom) and cells treated with Fc receptor blocking antibodies (Biolegend, United Kingdom). Cell suspensions were then labeled and sorted according to the specific protocol as below. All antibodies were purchased from Biolegend.

#### Mouse Lung Versus Aorta Endothelial Cells

Cells were stained with anti-CD45-PE/Cy7 (cluster of differentiation 45-phycoerythrin/cyanine 7), anti-CD31-AlexaFluor488, and anti-CD41-APC (allophycocyanin)/Cy7 and sorted using a FACSAria III instrument (BD Biosciences).

#### Mouse Lung Cell Panel

Cell suspensions were divided and labeled with one of the following antibody cocktails before sorting using a FACSMelody instrument (BD Biosciences). Antibody mix 1 (endothelial/epithelial/leukocyte): anti-epithelial cell adhesion molecule (EpCAM; CD326)-PE, anti-CD41-APC/Cy7, anti-CD45-PE/Cy7, anti-CD31-PerCP (peridinin chlorophyll protein)/Cy5.5, and anti-podoplanin-APC. Antibody mix 2 (mesenchymal): anti-EpCAM-PE, anti-CD41-PE, anti-CD45-PE, anti-Ter119 (Ter119 antigen)-PE, anti-CD31-PerCP/Cy5.5, anti-Sca1 (stem cell antigen-1)-PE/Cy7, anti-PDGFR (platelet-derived growth factor receptor), α-APC, and anti-CD9-APC/Fire750.

#### Human Lung Cell Panel

Cell suspensions were divided and labeled with one of the following antibody cocktails before sorting using a FACSMelody instrument (BD Biosciences). Antibody mix 1 (endothelial/epithelial): anti-CD41-APC/Cy7, anti-CD45-PerCP/Cy5.5, anti-CD31-FITC, anti-EpCAM-PE, and anti-podoplanin-APC. Antibody mix 2 (mesenchymal/leukocyte): anti-EpCAM-FITC, anti-CD31-FITC, anti-CD235a-FITC, anti-CD45-PerCP/Cy5.5, anti-CD41-APC/Cy7, and anti-CD146-PE.

#### Human Pulmonary Artery Endothelial Cells

Cells were stained with anti-CD41-APC/Cy7, anti-CD45-PerCP/Cy5.5, and anti-CD31-FITC and sorted using a FACSMelody instrument (BD Biosciences).

In each case, single live cells were identified from debris and doublets on the basis of scatter properties and negative DAPI (4′,6-diamidino-2-phenylindole) staining (Figure S6) and any cells bound to platelets excluded on the basis of CD41 staining. Populations of interest were identified using the gating strategies shown (Figure 4), and ≈10 000 cells were sorted using 2- or 4-way purity sort mode using a 100-µm nozzle. Fluorescence minus one controls were used to validate staining and define gating, which was further confirmed by qPCR for cell type marker gene expression in sorted populations (Table S3). After separation, cell populations were resuspended in DMEM media containing arachidonic acid (30 μM; Sigma) and incubated at 37 °C for 30 minutes. The release reaction was stopped by addition of diclofenac (10 μM; Sigma) and levels of the prostanoids measured in the supernatant by immunoassay as above and expressed relative to cell count.

**Figure 1.**
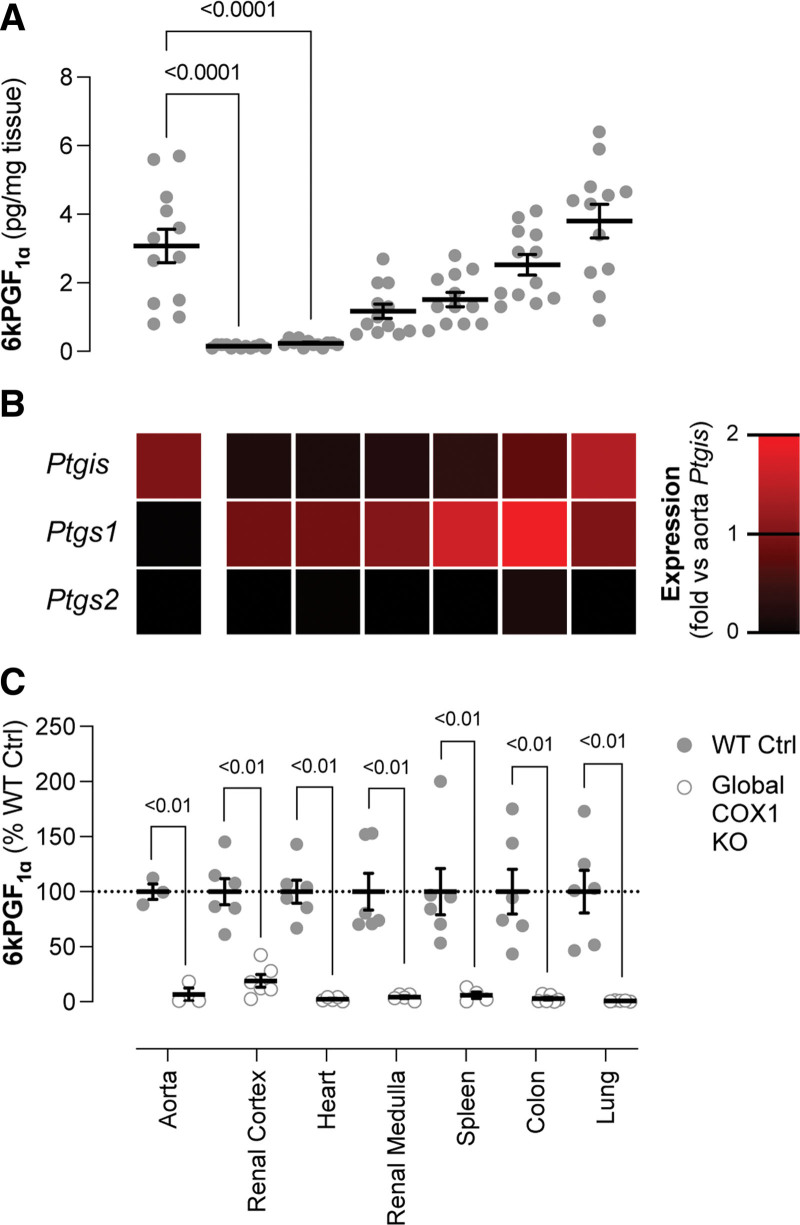
**Both tissues and arteries generate prostacyclin, which reflects relative prostacyclin synthase expression and requires COX (cyclo-oxygenase)-1. A**, Prostacyclin levels (measured as 6-keto prostaglandin F1α [6kPGF_1α_] after A23187 Ca^2+^ ionophore 30 μM stimulation) per unit mass (n=4) and (**B**) *Ptgis* (prostacyclin synthase), *Ptgs1* (COX-1) and *Ptgs2* (COX-2) gene expression (n=4) in wild-type mouse aorta and tissue. **C**, Prostacyclin release from tissues from global COX-1 knockout (global COX-1 KO) and matched wild-type control (WT Ctrl) mice (n=3–6). Data are mean±SEM with *P* values by Freidman test with Dunn post test (**A**) or unpaired *t* test (**C**) indicated where *P*<0.05. n is defined as the number of individual animals studied.

**Figure 2.**
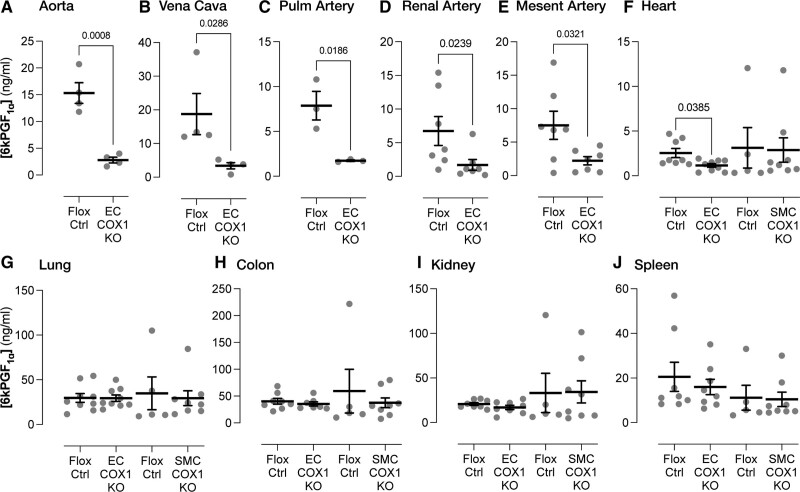
**Role of vascular COX (cyclo-oxygenase)-1 in prostacyclin release from arteries, veins, and tissues.** Prostacyclin release (measured as 6-keto prostaglandin F1α [6kPGF_1α_] after A23187 Ca^2+^ ionophore 30 μM stimulation) from (**A**) isolated aorta (n=4), (**B**) vena cava (n=4), (**C**) pulmonary artery (n=3), (**D**) renal artery (n=7), and (**E**) mesenteric artery (n=7) from endothelial COX-1 knockout (EC COX-1 KO) and Floxed littermate control animals (Flox Ctrl) and from intact segments of (**F**) heart (left ventricle; n=5–8), (**G**) lung (parenchyma; n=5–8), (**H**) colon (n=5–8), (**I**) kidney (renal medulla; n=5–8), and (**J**) spleen (n=5–8) from EC COX-1 KO mice, smooth muscle COX-1 knockout (KO), and their respective Floxed littermate controls (Flox Ctrl). Data are mean±SEM with *P* values by unpaired *t* test (**A**, **C**, **E**, **G**, **H**, and **I**) or Mann-Whitney *U* test (**B**, **D**, **F**, and **J**) indicated where *P*<0.05. n is defined as the number of individual animals studied.

**Figure 3.**
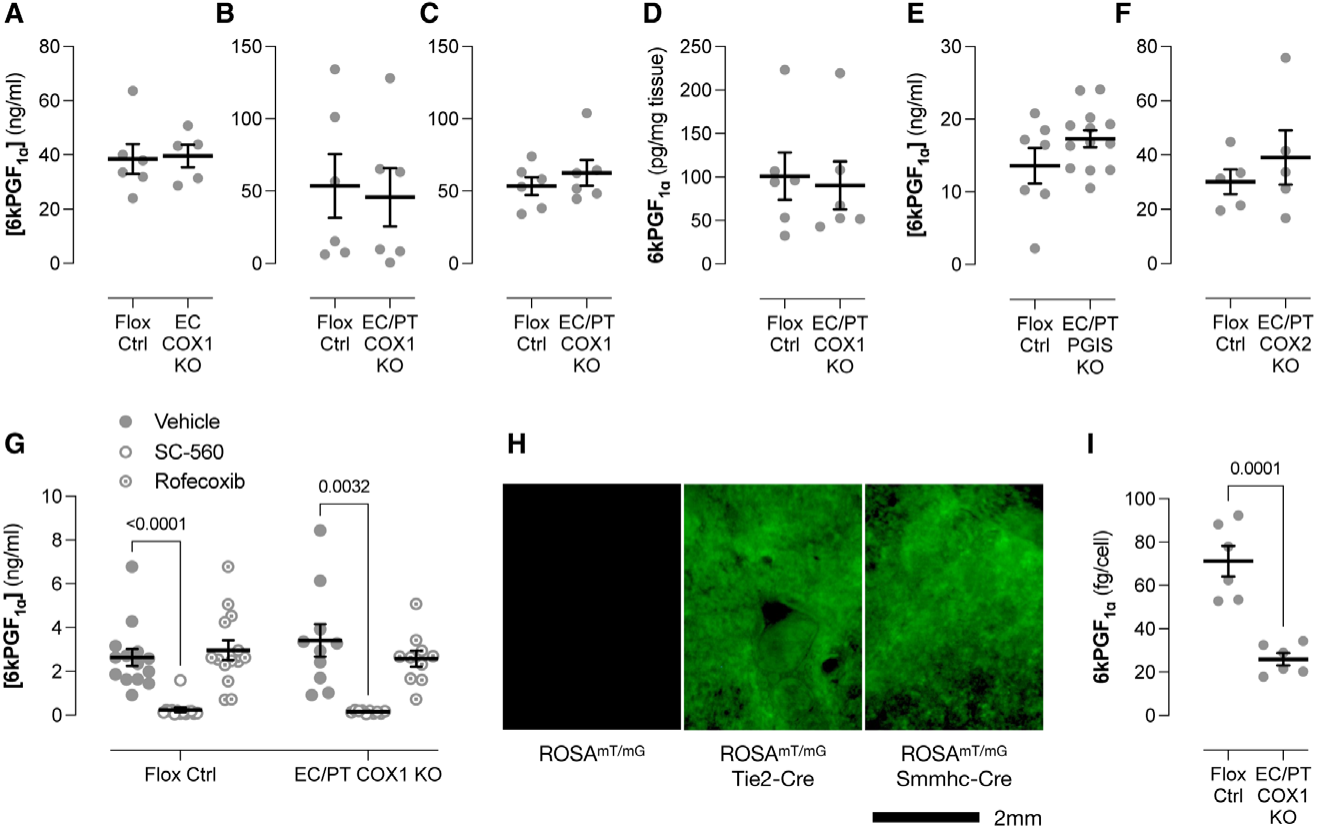
**Lung prostacyclin production does not require COX (cyclo-oyxgenase)-1, COX-2, or prostacyclin synthase in endothelial cells or platelets. A**, Prostacyclin release (measured as 6-keto prostaglandin F1α [6kPGF_1α_] after A23187 Ca^2+^ ionophore 30 μM stimulation) from lung parenchyma segments from endothelial COX-1 knockout (EC COX-1 KO) and Floxed littermate control animals (Flox Ctrl) in which the lung vasculature has been flushed of blood (n=5–6). **B**, Prostacyclin from lung parenchyma segments stimulated after stimulation with A23187 Ca^2+^ ionophore (n=6; 30 μM) or (**C**) arachidonic acid (n=6; 30 μM). **D**, Prostacyclin levels in unstimulated snap-frozen lung homogenates (n=6) from endothelial/platelet COX-1 knockout mice (EC/PT COX-1 KO). Prostacyclin release (A23187 Ca^2+^ ionophore 30 μM stimulation) from lung parenchyma segments from (**E**) endothelial/platelet prostacyclin synthase knockout mice (EC/PT PGIS KO; n=7–13) and (**F**) endothelial/platelet cyclo-oxygnease-2 knockout mice (EC/PT COX-2 KO; n=5), each compared with respective Floxed littermate controls. **G**, Prostacyclin release (A23187 Ca^2+^ ionophore 30 μM stimulation) from lung parenchyma segments from EC/PT COX-1 KO and Floxed littermate control animals (n=10–14) in the presence of selective inhibitors of COX-1 (SC-560; 1 µM) or COX-2 (rofecoxib; 1 µM). **H**, EGFP (enhanced green fluorescent protein; green) fluorescence in lung segments of ROSA^mT/mG^ mice with/without a *Tek*-Cre or *Tagln*-Cre transgene (representative of n=3 per genotype). **I**, Prostacyclin release from endothelial cells (CD31^+^, CD45^−^, CD41^−^) isolated by flow cytometry from EC/PT COX-1 KO and Flox Ctrl mouse lung (n=6). Data are mean±SEM with *P* values by unpaired *t* test (**A**, **B**, **C**, **E**, **F**, and **I**), Mann-Whitney *U* test (**D**), or 2-way ANOVA with Sidak post test (**G**) indicated where *P*<0.05. n is defined as the number of individual animals studied.

### Statistics and Data Analysis

Data are presented as mean±SE for n experiments. n refers to the number of independent biological replicates studied in any experiment—either individual animals, human donors, or primary cell lines established from tissue of separate donors. Where technical replicate measurements were made from the same individual, data were averaged before analysis. Samples from both male and female mice/donors were used, balanced across experimental groups in keeping with the ethical principles of animal and human research. Unless otherwise stated, data from both sexes were pooled and analyzed as a single group because studies were not powered to consider sex as an independent variable. Exploratory analyses (Figure S2) indicated no effect of sex on the underlying biology being studied; however, the combination of both data from male and female samples into a single group may be considered a limitation of our study design.

Statistical analysis was performed using the Prism 9 software (GraphPad Software) with the tests used indicated in individual figure legends. Normal distribution of each data set was determined using the Shapiro-Wilk test, and this was used to dictate the choice of parametric versus nonparametric statistical approach. Differences were considered significant where *P*<0.05. The corresponding author had full access to all the data in the study.

## RESULTS

### Tissues Are Major Sources of Prostacyclin Generated Through COX-1

Using the aorta as a benchmark, we first assayed prostacyclin formation per unit mass from paired mouse tissues to appreciate their relative capacity to generate prostacyclin. Prostacyclin release (stimulated by Ca^2+^ ionophore and measured as 6-ketoPGF_1α_) was observed from all tissues, with the lowest release from the renal cortex and heart and the highest release from the lung and colon (Figure 1A). Lung and colon produced equivalent prostacyclin to aorta on a per-mg-tissue basis, which, considering their large total mass, suggests they may be major contributors to whole body prostacyclin generation. The relative ability of tissues to generate prostacyclin broadly correlated with prostacyclin synthase gene (*Ptgis*) expression, which was enriched in aorta, colon, and lung relative to other tissues (Figure 1B; Table S1). By contrast, relative tissue levels of the COX-1 gene (*Ptgs1*) expression correlated poorly with prostacyclin release; for example, the aorta and lung expressed comparatively little *Ptgs1* gene (Figure 1B; Table S1). COX-2 (*Ptgs2*) was weakly expressed across all tissues (≈10-fold to 100-fold less than *Ptgs1*; Figure 1B; Table S1) in keeping with our previous observations of the relative constitutive expression and activity of the 2 COX isoforms.^12^ In agreement, global COX-1 (*Ptgs1*^−/−^) deletion abolished prostacyclin generation in all tissues studied (Figure 1C). This dominance of COX-1 in tissue prostacyclin generation in the systems studied cannot be explained by a loss of shear-maintained vascular COX-2 expression ex vivo^15^ because assays were completed within 1 hour postmortem and COX-2 protein has a half-life of >6 hours.^12^ Thus, arteries and tissues both generate prostacyclin, which is (1) driven by COX-1 activity but (2) at a gene expression level, reflects the relative level of prostacyclin synthase.

### Many Tissues Can Produce Prostacyclin in the Absence of Endothelial Cell COX-1

We next considered whether tissue prostacyclin release is simply a function of the constituent endothelial component or whether other cell types generate prostacyclin directly. To do this, we used mice in which COX-1 is specifically deleted from endothelial cells (*Ptgs1*^flox/flox^; VEC-Cre^ERT2^), which have been characterized previously.^4^ Aortic rings from these mice had marked reduction (≈80%) in prostacyclin release (Figure 2A) as did veins (Figure 2B) and arteries supplying the lung (Figure 2C), kidney (Figure 2D), and gut (Figure 2E). However, the effect of endothelial COX-1 deletion on prostacyclin release from isolated tissue segments was variable. In the heart, prostacyclin release when endothelial COX-1 is deleted was reduced ≈50%, suggesting a major role of endothelial COX-1 in prostacyclin in this tissue (Figure 2F). In contrast, in the lung (Figure 2G), colon (Figure 2H), kidney (Figure 2I), and spleen (Figure 2J), endothelial COX-1 deletion had no effect on prostacyclin release. The residual endothelium-independent tissue prostacyclin release was not accounted for by vascular smooth muscle COX-1 activity; smooth muscle COX-1 knockout mice (*Ptgs1*^flox/flox^; *Tagln*-Cre)^4^ exhibited no change in prostacyclin release in any tissue studied (Figure 2F through 2J). Thus, within most tissues, bulk prostacyclin production appears to be driven by nonendothelial, non–smooth muscle COX-1 activity. This was independent of sex because post hoc analysis indicated similar prostacyclin release by lung from male and female animals and no interaction between animal sex and the effect of endothelial COX-1 deletion (Figure S2). As such, follow-on studies used pooled data from both male and female animals/donors.

To validate and explore further these observations, we focused on the lung because (1) lung has the highest prostaglandin production among tissues (Figure 1A), (2) lung prostacyclin production appeared to be almost completely independent of vascular endothelial/smooth muscle COX-1 (Figure 2G), and (3) the anatomic position of the lung means prostacyclin generated here is likely to directly influence the heart and major arteries.^22^ We first considered whether there may be other sources that can donate PG H_2_ to endothelial cells for conversion to mature prostacyclin, bypassing the effect of endothelial COX deletion. This phenomenon of transcellular metabolism has been described previously in platelet/endothelial cocultures where platelet-derived PG H_2_ can enter endothelial cells to access prostacyclin synthase.^39^ However, even after flushing the pulmonary vasculature of blood to remove circulating platelets, endothelial COX-1 deletion had no effect on prostacyclin release (Figure 3A). We further confirmed this result by studying dual endothelial/platelet COX-1 knockout mouse (*Ptgs1*^flox/flox^; *Tek*-Cre) where PG H_2_ cannot be synthesized either by platelets or by endothelial cells.^4^ As observed in endothelial-specific COX-1 knockout mice, endothelial/platelet COX-1 deletion had no effect on lung tissue release of prostacyclin stimulated by Ca^2+^ ionophore (Figure 3B) or exogenous arachidonic acid (Figure 3C). While valuable in determining gross synthetic capacity, ex vivo mediator release assays have a potential to produce artifactual results due to loss of the in vivo environment, use of exogenous stimuli, and removal from normal metabolic/excretion pathways. We, therefore, considered whether these patterns of prostacyclin generation seen in ex vivo release assays corresponded to production in vivo. Measuring prostanoid formation in vivo is complex, with different approaches favored by different researchers and no universally agreed upon techniques. We took the approach of measuring prostacyclin levels in snap-frozen lung tissue, cold homogenized in an excess of COX inhibitor to prevent ex vivo prostanoid generation. In this system, lung prostacyclin levels (as 6-ketoPGF_1α_) were detected at modest levels of ≈100 pg/mg tissue (Figure 3D), which compares well with the findings of others using mass spectrometry–based methods.^40^ Using this approach, we confirmed no effect of endothelial/platelet COX-1 deletion on in vivo lung prostacyclin levels (Figure 3D). To exclude a role for other pathways of PG H_2_ generation, we studied lung from endothelial/platelet prostacyclin synthase knockout mice (Figure 3E; *Ptgis*^flox/flox^; *Tek*-Cre) and endothelial/platelet COX-2 knockout mice (Figure 3F; *Ptgs2*^flox/flox^; *Tek*-Cre). Lung from both strains retained a full capacity to generate prostacyclin, confirming that endothelial cells in the lung are neither required to generate PG H_2_ nor to convert PG H_2_ from other sources into prostacyclin. Data from these mouse knockout models also corroborated the relative role of endothelium in prostacyclin release in other tissues; these are presented in Table S2 but not discussed further. To extended this, we established whether nonendothelial prostacyclin release could be mediated by cooperativity or redundancy from COX-2 when endothelial COX-1 was disrupted. Selective COX-2 inhibition by rofecoxib had no impact on prostacyclin release from endothelial/platelet COX-1 knockout lung tissue (Figure 3G). By contrast, global COX-1 inhibition by SC-560 abolished prostacyclin release (Figure 3G) in agreement with our data from global COX-1 knockout mouse tissue (Figure 1C). As such, we focused on COX-1–mediated pathways for subsequent experiments to understand nonendothelial prostacyclin generation.

We next explored whether retention of prostacyclin generation by the lung in these models might be due to incomplete penetrance of Cre-mediated recombination in tissue versus arterial endothelial cells. Previous studies have effectively used *Tek*-Cre^41^ and VEC-Cre^42^ to delete Floxed genes in lung microvasculature. To confirm this, we crossed *Tek*-Cre mice (used to generate endothelial/platelet COX-1, COX-2, and prostacyclin synthase knockout mice) and *Tagln*-Cre mice (used to generate smooth muscle COX-1 knockout mice) with an EGFP reporter strain. Robust recombination occurred throughout the lung (Figure 3H). We went on to isolate live microvascular endothelial cells from the lung of endothelial/platelet COX-1 knockout mice by fluorescence-activated cell sorting (FACS) and found ≈70% reduction in prostacyclin production compared with cells isolated from the lung of Floxed littermate control mice; these data confirm effective loss of COX-1 activity in lung endothelium (Figure 3I). Finally, to ensure that 6-ketoPGF_1α_ detected by immunoassay represented release of genuine bioactive prostacyclin, we used a human platelet bioassay,^4^ analogous to the methodology used in the original identification of prostacyclin.^1^ These experiments confirmed that global COX-1 deficiency/inhibition, but not endothelium-specific or endothelial/platelet COX-1 deletion, resulted in loss of prostacyclin-like activity released from the lung in full agreement with idea that lung prostacyclin generation occurs independently of the endothelial COX-1/prostacyclin synthase pathway (Figure S3).

### Endothelial Cells From the Lung Are Deficient in Prostacyclin Synthesis Compared With Those From Large Arteries

**Figure 4.**
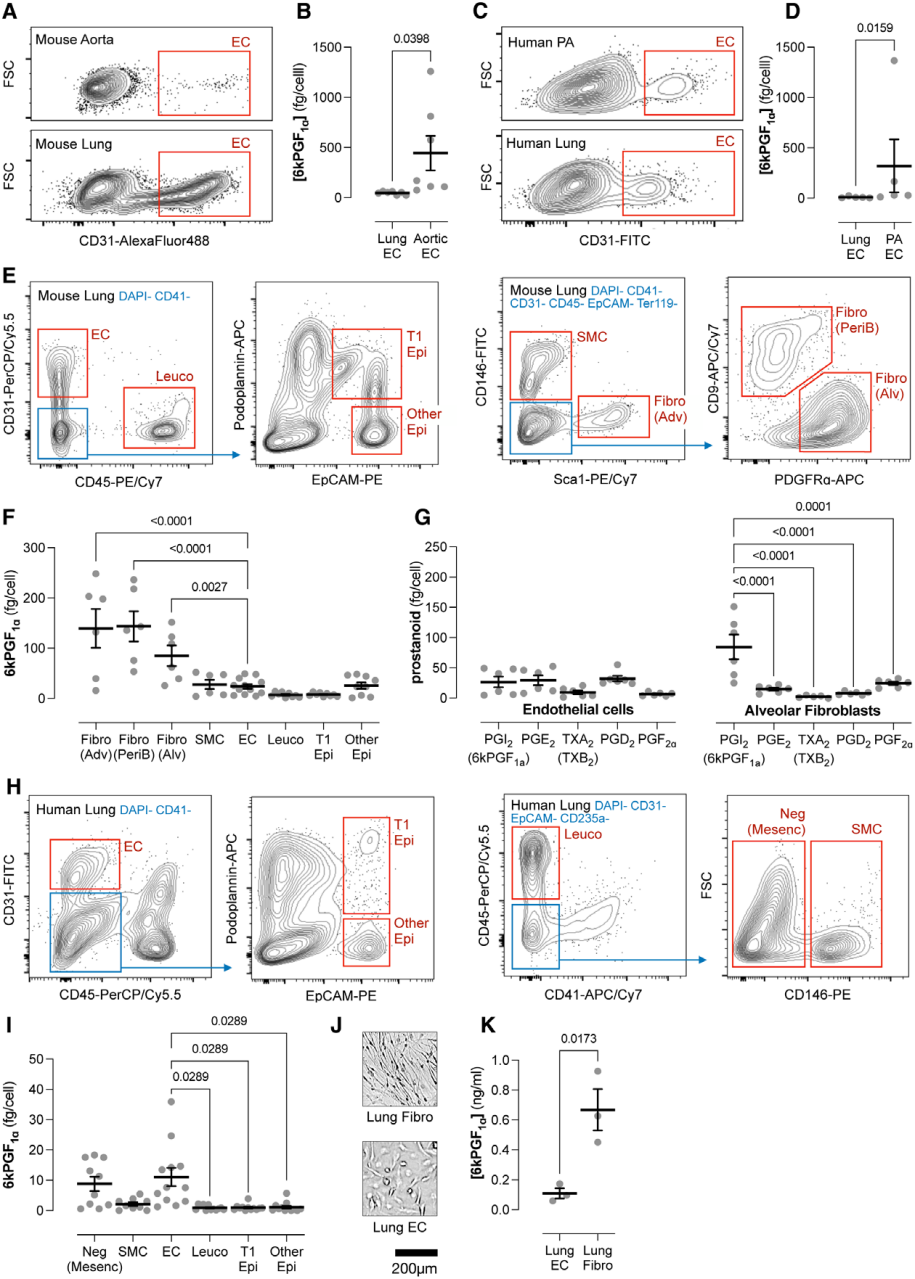
**Fibroblasts are major contributors to lung prostacyclin generation. A**, Fluorescence-activated cell sorting (FACS) gating strategy (representative plots) and (**B**) prostacyclin release (measured as 6-keto prostaglandin F1α [6kPGF_1α_] after arachidonic acid 30 μM stimulation; n=4) of endothelial cells from matched mouse aorta and lung parenchyma. **C**, FACS gating strategy (representative plots) and (**D**) prostacyclin release (n=5 donors) of endothelial cells from matched human pulmonary artery and lung. **E**, FACS gating strategy (representative plots) and (**F**) prostacyclin release from endothelial cells (EC), leucocytes (Leuco), type 1 (T1 Epi), and other epithelial cells (Other Epi), smooth muscle cells (SMCs) and adventitial (Fibro Adv), alveolar (Fibro Alv), and peribronchial fibroblasts (Fibro PeriB) from mouse lung (n=6–12). **G**, Relative release of primary prostanoids from fresh FACS isolated lung endothelial and alveolar fibroblasts (n=6). **H**, FACS gating strategy (representative plots) and (**I**) prostacyclin release from EC, Leuco, T1 Epi, Other Epi, SMC, and negatively selected mesenchymal cells (Neg Mesenc) from human lung (n=9–12 donors). **J**, Representative bright-field images and (**K**) prostacyclin release (after arachidonic acid 30 μM stimulation; n=3 donors) from cultured primary human lung microvascular endothelial cells (Lung EC) and human lung fibroblasts (Lung Fibro). FACS plots show 5% density contours. Data are mean±SE with *P* values by unpaired *t* test (**B** and **K**), Mann-Whitney *U* test (**D**), or repeated measures 1-way ANOVA with Holm-Sidak post test (**F**, **G**, and **I**) indicated where *P*<0.05. n is defined as the number of individual animals (**B**, **F**, and **G**), human donors (**D** and **I**), or independent donor primary cell lines (**K**) studied.

The lung is among the most highly vascularized organs in the body; therefore, to understand the presence of a nonendothelial prostacyclin pathway, we had to first determine why lung endothelial cells do not meaningfully contribute to prostacyclin production. Lung microvascular endothelial cells have previously been observed to possess an altered prostanoid synthesis profile compared with arterial endothelial cells, particularly elevated prostaglandin E_2_ formation.^29,43^ However, to our knowledge, freshly isolated lung microvascular and arterial endothelial cells have not been compared head to head for their relative ability to synthesize prostacyclin. Therefore, to address this issue, we sorted fresh, matched endothelial cells (CD31^+^, CD45^−^, CD41^−^) from mouse aorta and lung by FACS (Figure 4A) and tested their ability to release prostacyclin immediately after isolation. Endothelial cells from the lung released prostacyclin at much lower levels compared with those from the aorta (Figure 4B) in agreement with the broader idea that endothelial cells from the lung microvasculature carry a more immature, stem-like phenotype in comparison to endothelial cells from arteries and veins.^44^ Moreover, single-cell RNA sequencing studies of endothelial heterogeneity identified prostacyclin synthase (*PTGIS*) in the top 20 transcripts differentiating human lung arterial and capillary endothelial cells.^45^ To translate our findings from mice, we obtained matched, histologically normal pulmonary artery and lung parenchyma from human donors undergoing lung resection for carcinoma and isolated endothelial cells from each using the same approach (Figure 4C). As observed in mice, endothelial cells from human lung parenchyma showed the same pattern of lesser prostacyclin synthesis when compared with endothelial cells isolated from pulmonary arteries of the same individuals (Figure 4D).

### Fibroblasts Are the Principal Contributors to Prostacyclin Production in the Lung

If endothelial cells are not the major source of lung prostacyclin, what cell types account for its production? This question cannot be addressed by immunohistochemical/gene expression approaches, both because of the complex cascade of enzymes and biochemical factors required to support prostacyclin synthesis and because of limitations in the specificity/sensitivity of antibodies. Therefore, we again studied cell populations rapidly isolated by FACS from fresh mouse lung tissue. Using endothelial cells (CD31^+^ CD45^−^) as a benchmark, we profiled prostacyclin release from epithelial cells (EpCAM^+^, podoplanin^−^), type I alveolar epithelial cells (EpCAM^+^, podoplanin^+^), and leucocytes (CD45^+^; Figure 4E) as described previously.^46^ We also isolated a mesenchymal cell population by exclusion (EpCAM^−^, CD45^−^, CD31^−^, Ter119^−^) and, within this, used an approach defined from single-cell RNA-seq analysis of the lung^47^ to select for SMCs (CD146^+^), adventitial fibroblasts (CD146^−^ Sca1^+^), alveolar fibroblasts (CD146^−^ Sca1^−^ PDGFRα^+^), and peribronchial fibroblasts (CD146^−^, Sca1^−^, PDGFRα^−^, CD9^+^; Figure 4E). When stimulated with arachidonic acid, leucocytes, epithelial cells, and SMCs released comparable (and numerically less) prostacyclin to endothelial cells (Figure 4F). However, fibroblasts exhibited significantly greater prostacyclin production (up to 6-fold greater than endothelial cells; Figure 4F). Importantly, this was true of each type of fibroblast studied including peribronchial, alveolar, and adventitial fibroblasts and indicated that within the lung, prostacyclin may be generated by fibroblasts in the lung both within and outside the vascular wall. This ability of fibroblasts to generate prostacyclin was selective, as release of other prostanoids (TXA_2_, prostaglandin E_2_, prostaglandin F_2α_, and prostaglandin D_2_) was low and comparable to endothelial cells (Figure 4G). To exclude the possibility that the prostacyclin-generating capacity associated with fibroblast fractions represented contamination with other cell types, we performed additional characterization of FACS fractions by RT-qPCR. We found the expression of the endothelial cell marker, VEC (*Cdh5*), was restricted to the endothelial cell fraction and expression of the SMC and pericyte markers, α-smooth muscle actin (*Acta2*), neural/glial antigen-2 (*Cspg4*), and PDGFRβ (*Pdgfrb*), restricted to the SMC fraction (Table S3). The 3 fibroblast fractions showed little to no expression of any of the above markers, confirming their purity (Table S3). These data, therefore, support the hypothesis that lung endothelial cells, smooth muscle cells, pericytes, and leucocytes are poor producers of prostacyclin in comparison to lung fibroblasts (both vascular and nonvascular). It cannot be excluded there may also be roles for other niche vascular/nonvascular cell types that we have not considered.

We replicated these studies with cells isolated from fresh, histologically normal human lung tissue. Endothelial cells (CD31^+^ CD45^−^), epithelial cells (EpCAM^+^, podoplanin^−^), type I alveolar epithelial cells (EpCAM^+^, podoplanin^+^), and leucocytes (CD45^+^) were defined and isolated in the same fashion as from mouse lung (Figure 4H). Because suitable cell surface markers to positively select human lung fibroblast populations have not been defined, we studied only a negatively selected mesenchymal cell population (EpCAM^−^, CD45^−^, CD31^−^, CD235a^−^) that was subdivided into SMCs (CD146^+^) and other cells (CD146^−^; Figure 4H). Prostacyclin release from leukocytes, epithelial cells, and smooth muscle cells was low, when compared with endothelial cells (Figure 4I). Prostacyclin levels released from negatively selected mesenchymal cells were similar to those from endothelial cells (Figure 4I). These data are consistent with the suggestion that, in human lung, both fibroblasts and endothelial cells are contributors to total prostacyclin release. However, because this negative selection approach may underestimate fibroblast prostacyclin synthesis as a consequence of contamination by other cell types, we performed similar experiments using commercially sourced primary human lung cell cultures (Figure 4J). In carefully matched experiments where primary cells of similar and low passage were grown under identical conditions and stimulated with arachidonic acid, prostacyclin levels released from primary human lung fibroblasts were greater than those from primary lung microvascular endothelial cells (Figure 4K). Although these data are interpreted with some caution given the changes in prostanoid pathways that can occur in vitro,^48^ the results are fully supportive and in agreement with our findings from freshly isolated mouse and human cells that fibroblasts are central contributors to lung prostacyclin production.

**Figure 5.**
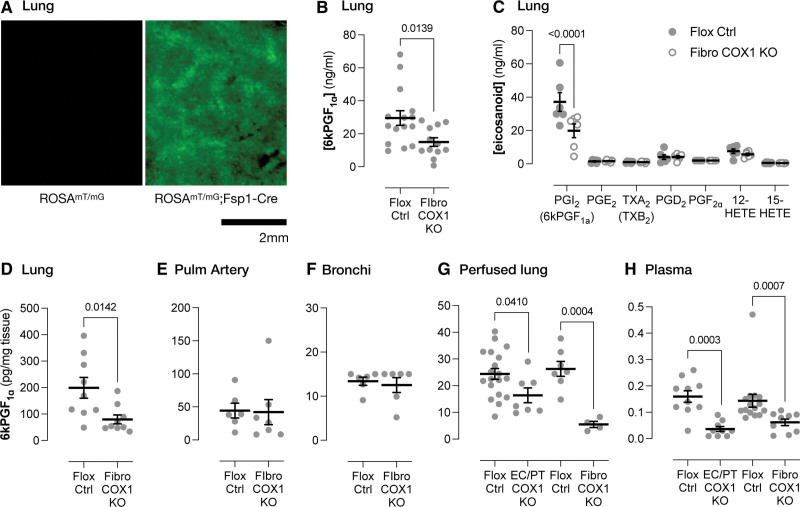
**Fibroblasts contribute to total lung prostacyclin production and from where it can enter the systemic circulation. A**, EGFP (enhanced green fluorescent protein) fluorescence (green) in lung segments of ROSA^mT/mG^ mice with/without a Fsp1 (fibroblast-specific protein-1)-Cre transgene (representative of n=3 per genotype). Release (A23187 Ca^2+^ ionophore 30 μM stimulation) by lung parenchyma segments of (**B**) prostacyclin (n=12–16) and (**C**) a panel of primary prostanoids and HETEs (n=6) from fibroblast-specific COX (cyclo-oxygenase)-1 knockout (Fibro COX-1 KO) and Floxed littermate control mice (Flox Ctrl). **D**, Prostacyclin levels in unstimulated snap-frozen lung homogenates from fibro COX-1 KO knockout mice and Flox Ctrl animals (n=9–10). Prostacyclin release (A23187 Ca^2+^ ionophore 30 μM stimulation) from (**E**) pulmonary artery (n=6–7) and (**F**) bronchi from fibro COX-1 KO knockout mice and flox Ctrl animals (n=6–7). Prostacyclin levels (measured as 6-keto prostaglandin F1α [6kPGF_1α_]) measured in the outflow from (**G**) isolated perfused lung (n=4–19) and (**H**) in plasma (n=9–15) from endothelial/platelet COX-1 knockout (EC/PT COX-1 KO), fibro COX-1 KO, and respective Flox Ctrl mice. Data are mean±SE with *P* values by Mann-Whitney *U* test (**B**, **D**, **E**, **F**, **H**) or repeated measures 2-way ANOVA with Holm-Sidak post test (**C**) or unpaired *t* test (**G**) indicated where *P*<0.05. n is defined as the number of individual animals studied.

Having identified fibroblasts as the major prostacyclin-producing cells in the mouse and human lung, we returned to a mouse cell-specific knockout approach to understand the fibroblast contribution to bulk tissue prostacyclin release. We generated a fibroblast COX-1 knockout model driven by Cre expression from the Fsp1/*S100a4* promoter, which is expressed in lung fibroblasts.^49^ Although this promoter is also active in monocyte/macrophages, these cells have almost no prostacyclin synthetic capacity (Figure 4F and 4I), suggesting there should be little consequence of off-target deletions here. To confirm the suitability of this model, we crossed the Fsp1-Cre mice with EGFP reporter animals and observed robust recombination within the lung (Figure 5A). When Fsp1-Cre mice were crossed onto a Ptgs1^flox/flox^ background to generate fibroblast-specific COX-1 knockout mice (*Ptgs1*^*flox/flox*^*;Fsp1-Cre*), we observed a loss of COX-1 mRNA in lung-derived fibroblasts but full retention of COX-1 mRNA in the aorta (Figure S1). An ≈50% reduction in stimulated prostacyclin release was seen from intact segments of lung parenchyma from these mice (Figure 5B), and this was accompanied by a similar reduction in total lung COX-1 mRNA (Figure S1). Post hoc analysis indicated that the effect on prostacyclin release was independent of animal sex (Figure S2). In agreement with our data from isolated lung cells, this effect was selective to prostacyclin; release of the other primary prostanoids (prostaglandin E_2_, TXA_2_, prostaglandin D_2_, and prostaglandin F_2α_) and the related eicosanoids 12-HETE and 15-HETE were not altered from fibroblast COX-1 knockout mouse lung (Figure 5C). Data from these ex vivo release experiments reflected production in vivo with prostacyclin levels in snap-frozen lung homogenates reduced by fibroblast-specific COX-1 deletion (Figure 5D). These data support our observations from mouse and human cells and the hypothesis that fibroblasts are major contributors to lung tissue prostacyclin production. Because lung prostacyclin release is abolished by global COX-1 deletion (Figure 1C) or inhibition (Figure 3G), the residual prostacyclin synthesis observed in these mice is unlikely to be accounted for by COX-2 and instead may be attributed to COX-1 in other cell types or subsets of fibroblasts that do not express the Fsp1-Cre transgene. Fibroblast-specific COX-1 deletion also showed a strong trend to reduce prostacyclin release from gut tissue (colon; *P*=0.07), which, like the lung, exhibited prostacyclin release essentially independent of the endothelial COX-1 (Table S4). No effect of fibroblast-specific COX-1 knockout on prostacyclin release was observed from pulmonary artery (Figure 5E), bronchi (Figure 5F), aorta, heart, kidney, or spleen (Table S4).

### Nonendothelial Prostacyclin Can Act Systemically to Reduce Antithrombotic Tone

To consider the biological significance of prostacyclin produced outside the vascular wall, we explored whether the prostacyclin produced by fibroblasts in tissues acts simply as a local lung mediator or whether it can produce systemic effects either by entering the circulation or acting on circulating cells as they pass through organs. To do this, we used an ex vivo isolated perfused lung preparation in which released prostacyclin is collected through the organ’s vasculature. In this model, prostacyclin in the perfused tissue effluent (detected as its hydrolysis product 6-ketoPGF_1α_) was reduced in both endothelial/platelet COX-1 knockout and fibroblast-specific COX-1 knockout lung (Figure 5G). To establish whether this also occurs in vivo, we measured the levels of 6-ketoPGF_1α_ in plasma. Although some have questioned the validity of immunoassay approaches to measurement of prostacyclin in complex biological fluids,^50^ we have previously validated our ELISA technique against mass spectrometry and found direct quantitative equivalence in mouse plasma^8^ and separately shown that the immunoreactive 6-ketoPGF_1α_ in mouse plasma is completely lost by specific deletion of prostacyclin synthase^51^ or COX-1.^4^ Using this same approach, we found plasma 6-ketoPGF_1α_ levels to be reduced in plasma from both endothelial/platelet and fibroblast COX-1 knockout mice, when compared with their respective control strains (Figure 5H). Thus, prostacyclin derived from both endothelial cells and prostacyclin derived from COX-1 in fibroblasts can be detected as 6-ketoPGF_1α_ in the vascular compartment. This implies prostacyclin generated in the lung has the potential to exert systemic effects, either by brief circulation of the vasculature in a bioactive form (which is plausible but a controversial idea^52,53^) or by acting locally on platelets and circulating cells as they pass through the lung circulation.

**Figure 6.**
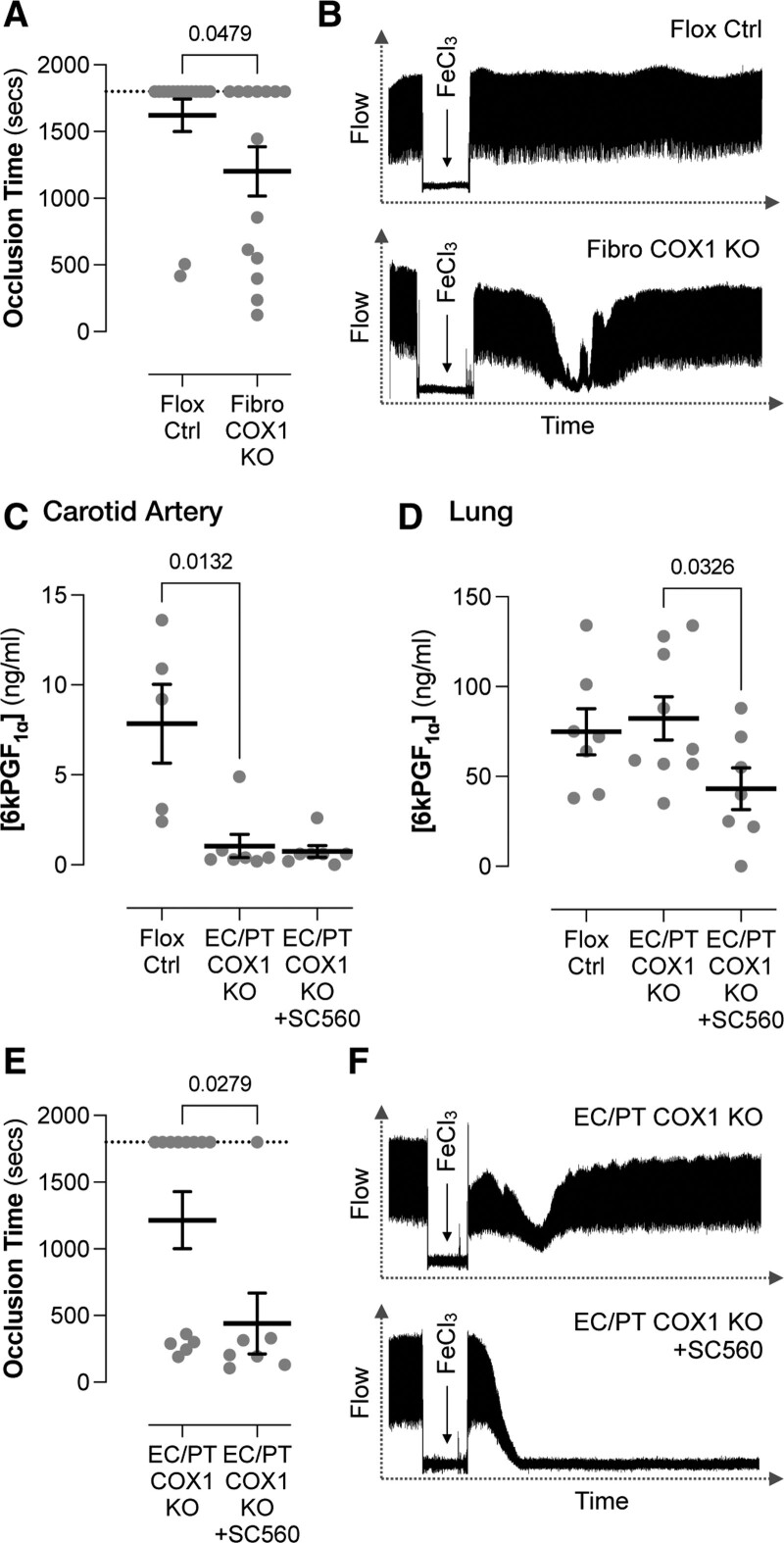
**Fibroblasts and other nonendothelial sites of COX (cyclo-oxygenase)-1 expression contribute to systemic antithrombotic protection. A**, Thrombotic occlusion time (n=14–15) and (**B**) representative blood flow traces after carotid artery FeCl_3_ injury in vivo in fibroblast COX-1 knockout mice (Fibro COX-1 KO) and Floxed littermate controls (Flox Ctrl). Prostacyclin release (measured as 6-keto prostaglandin F1α [6kPGF_1α_]) from (**C**) carotid artery (n=7) and (**D**) lung parenchyma (n=7–9) ex vivo from endothelial/platelet COX-1 knockout mice (EC/PT COX-1 KO) treated with the COX-1 inhibitor, SC-560 (10 mg/kg; IV; 15 min), or vehicle (5% dimethyl sulphoxide). Release level from Flox Ctrl tissue is marked on each graph as a dashed line. **E**, Thrombotic occlusion time (n=7–13) and (**F**) representative blood flow traces after carotid artery FeCl_3_ injury in vivo in EC/PT COX-1 KO treated with SC-560 or vehicle. Data are mean±SE with *P* values by Mann-Whitney *U* test (**A** and **E**) or Kruskal-Wallis test with Dunn post test (**C**) or 1-way ANOVA with Holm-Sidak post test (**D**) indicated where *P*<0.05. n is defined as the number of individual animals studied.

To address this, we determined whether nonendothelial prostacyclin depots contribute to cardiovascular protection in the same way we understand for endothelium-derived prostacyclin. This was particularly important given only a partial lung prostacyclin reduction was observed in fibroblast-specific COX-1 knockout mice. The biological effects of partial prostacyclin deficiency have not been fully explored, but it is important to note that we have found heterozygous deletion of prostacyclin synthase is sufficient to exacerbate renal ischemia perfusion injury in mice^28^ and others have found heterozygous prostacyclin receptor mutations in man are associated with increased atherothrombotic risk.^5^ Prostacyclin is best understood as an antiplatelet/antithrombotic factor, balancing the action of platelet-derived thromboxane. We have previously used an in vivo FeCl_3_ carotid artery injury model to demonstrate a prothrombotic phenotype in endothelium-specific COX-1 knockout mice^4^ and an antithrombotic phenotype in platelet-specific or dual endothelial/platelet COX-1 knockout mice.^4,54^ Here we used the same model to show that fibroblast-specific COX-1 knockout mice exhibit a modest but significant prothrombotic phenotype (Figure 6A and 6B). This result was not associated with any change in local carotid artery prostacyclin generation (Flox Ctrl: 33.1±9.5 ng/mL; Fibro COX-1 KO: 34.5±23.3 ng/mL; n=6; *P*>0.05 by unpaired *t* test), which we have previously shown to be predominately generated by endothelial cells.^4^ We next considered whether there may be other sources that contribute to the total antithrombotic contribution of all nonendothelial COX-1–mediated prostacyclin generation in the body that are not accounted for by fibroblast-specific deletion. To address this limitation, we treated endothelial/platelet COX-1 knockout mice with the selective COX-1 inhibitor, SC-560, to determine the effect of removal of all residual COX-1–derived prostanoids. Because these mice already lack COX-1 in platelets and endothelial cells, no effect of SC-560 on carotid artery prostacyclin levels (Figure 6C) or platelet thromboxane levels was noted (Figure S4) but prostacyclin release by lung tissue was reduced ≈50% (Figure 6D). This was associated with a marked increase in thrombosis after carotid artery FeCl_3_ injury (Figure 6E and 6F). This effect could not be attributed to an off-target SC-560 effect, because SC-560 had no effect on thrombosis in global COX-1–deficient mice (Figure S5). These data support the idea that inhibitory prostanoids derived from COX-1 in fibroblasts and other nonendothelial sources contribute to systemic antithrombotic protection. This may occur either through fibroblast-derived prostanoids directly entering the vascular compartment and circulating to sites of thrombosis or by conditioning platelets as they pass through the vasculature of the lung and other organs. It should be noted that it cannot be unequivocally proven that this effect is mediated by prostacyclin because models of fibroblast prostacyclin synthase deletion would exhibit shunting of excess PG H_2_ into other prostanoid products.^51^ However, given that prostacyclin is the major antithrombotic prostanoid species and that prostacyclin is the most abundant prostanoid produced by fibroblasts (Figure 4G), it is logically the most likely candidate to mediate the antithrombotic role of fibroblast COX-1. Nonetheless, roles for other prostanoids such as prostaglandin D_2_ in this effect cannot be definitively excluded.

## DISCUSSION

The identification of prostacyclin was rapidly followed by the idea that its manipulation could offer the means to prevent and treat cardiovascular disease.^1,2^ Since its discovery, prostacyclin has been mostly commonly associated with the endothelium. Our current findings suggest, however, a substantive role for fibroblasts in both COX-1–mediated prostacyclin synthesis and systemic antithrombotic protection. While prostacyclin production by fibroblasts and other nonendothelial sources is not in itself a novel concept, this report demonstrates that these other depots of prostacyclin production make a meaningful and functionally significant contribution to prostacyclin’s systemic bioactivity. Indeed, while isolated fibroblasts from several tissues have been shown previously to have the ability to produce prostacyclin in vitro,^24–26^ we have now demonstrated the unappreciated extent to which this contributes to total prostacyclin generation and systemic biological effect in vivo. Thus, we must now consider the biological role and therapeutic potential of nonendothelial prostacyclin in the lung and elsewhere:

First, it is clear that pulmonary vascular disease is associated with prostacyclin deficiency^55^ and cardiovascular risk. These new findings raise the possibility that diseases of the lung parenchyma may also be associated with a loss of cardioprotective prostacyclin. For example, both chronic obstructive pulmonary disorder and interstitial lung disease feature dysfunction, damage, and phenotypic alterations to a range of lung parenchymal cells, including fibroblasts. An impairment of the ability of these cells to produce prostacyclin could not only contribute to disease pathogenesis but also explain the increased atherothrombotic risk observed in both conditions.^56,57^ Indeed, lung tissue from patients with chronic obstructive pulmonary disorder exhibited reduced prostacyclin synthase expression,^58^ and fibroblasts isolated from pulmonary fibrosis patients show reduced prostacyclin and increased thromboxane formation.^59^ These data suggest that a prostacyclin-based therapy may help to mitigate the excess cardiovascular risk and treat disease progression in these specific patient groups. In support of this suggestion, it has recently been found that inhaled trepostinil has therapeutic benefits in the treatment of idiopathic pulmonary fibrosis.^60^

Second, our finding that lung microvascular endothelial cells in vivo are relatively deficient in prostacyclin production may also suggest an opportunity to boost its endogenous generation to treat both lung and thrombotic disease. Lung overexpression of prostacyclin synthase is protective including in models of pulmonary hypertension,^61^ and while this nonspecific delivery is clearly effective, in light of our findings, we suggest that, to maximize the efficacy and reduce the systemic side effects, prostacyclin synthase delivery approaches would best be targeted to the pulmonary endothelium. Recently developed simple polymer-based transfection reagents that selectively deliver mRNA cargoes to lung endothelium^62^ could provide a practical route to achieving this.

Third, we must also consider the possibility that fibroblast-derived prostacyclin may have important autocrine or paracrine roles. Prostacyclin has well-defined effects on lung cell function including bronchodilation, immunomodulation, and inhibition both of fibrosis and proliferation. For example, we have previously shown that COX-1–derived prostanoids regulate airway function^63^ and others have shown roles for endogenous prostacyclin in lung fibrosis after bleomycin-induced lung injury^64^ and in regulating intravascular thrombosis in pulmonary hypertension.^65^ All these responses may be associated, partly or wholly, with prostacyclin derived from nonendothelial sources, which should now be considered. While it has not been the focus of the current study, it should be remembered that in settings of disease^64^ or discrete local niches,^66^ there may be equally important roles for prostanoids derived from COX-2 in fibroblasts or other cell types.

The previously unappreciated degree to which nonendothelial sources contribute to prostacyclin generation presents a new concept. This is not suggested to reduce emphasis on endothelial COX-1^4^ and COX-2–derived prostacyclin^4^ and the consequences of these powerful antithrombotic pathways—the evidence of their importance is clear. It should also not be forgotten that prostacyclin signaling pathways may be activated by nontraditional ligands including 12-HETE,^67^ other prostanoids,^68^ and molecules derived from other fatty acid substrates.^69,70^ Our findings, however, suggest we must now consider an additional, parallel cardioprotective or local disease-modifying pathway associated with COX-1 and prostacyclin outside the vascular endothelium and smooth muscle. In principle, each of these pathways results in activation of the same prostacyclin receptor signaling cascades, but there appears to be a lack of redundancy such that each of these alternative sources of prostacyclin synthesis and receptor activation carries unique biological functions. Consequently, loss of any individual prostacyclin synthesis/activation pathway may have consequences for cardiovascular health. The roles of these, individually and together, should now be evaluated in context, through health and disease and across organ systems. The results suggest new therapeutic opportunities within these new prostacyclin pathways, for the treatment of a range of human diseases, including cardiovascular disease as a comorbidity of respiratory conditions.

## ARTICLE INFORMATION

### Acknowledgments

The authors acknowledge Dr Jess Rowley, Dr Larissa Zárate García, and the Imperial College South Kensington Flow Cytometry Facility for their assistance in flow cytometry, Harshil Bhayani and the Royal Brompton and Harefield NHS (National Health Service) Trust Biomedical Research Unit Advanced Lung Disease Biobank for their assistance in obtaining human lung tissue and Prof Timothy Warner for his provision of global cyclo-oxygenase-1–deficient mice.

### Sources of Funding

The work was supported by grants from the British Heart Foundation (FS/16/1/31699 to N.S. Kirkby; RG/18/4/33541 and FS/4yPhD/F/21/34165 to J.A. Mitchell and N.S. Kirkby), Imperial College/Wellcome Trust (to B. Ahmetaj-Shala), the National Institutes of Health/National Cancer Institute (R01-CA084572, R01-CA123055, and P50-CA086306 to H.R. Herschman), the Phelps Family Foundation and the Crump Family Foundation (to H.R. Herschman), the National Natural Science Foundation of China (81770678 to B. Liu and 31771272 to Y. Zhou), and the Natural Science Foundation of Guangdong Province (2019A1515011650 to B. Liu).

### Disclosures

J.A. Mitchell is a shareholder of and member of the scientific advisory board for Antibe Therapeutics, which develops cyclo-oxygenase inhibitor anti-inflammatory drugs. J.A. Mitchell and N.S. Kirkby hold active research grant funding in the area of cyclo-oxygenase biology. The other authors report no conflicts.

### Supplemental Material

Tables S1–S4

Figures S1–S6

Major Resources Table

## Supplementary Material


