# Supplementary material for: Widening the Prostacyclin Paradigm: Tissue Fibroblasts Are a Critical Site of Production and Antithrombotic Protection
Source: Arterioscler Thromb Vasc Biol. 2023 Oct 12;44(1):271–86. doi: 10.1161/ATVBAHA.123.318923 (PMC10749679; doi:10.1161/ATVBAHA.123.318923)
Supplement: Supplementary file 1 [file atv-44-271-s001.pdf]

Widening the prostacyclin paradigm: tissue fibroblasts are a critical site of production and anti-thrombotic protection

Authors:

Maria Vinokurova[1#], Maria Elisa Lopes-Pires[1#], Neringa Cypaite[1], Fisnik Shala[1], Paul C. Armstrong[2], Blerina Ahmetaj-Shala[1], Youssef Elghazouli[1], Rolf Nüsing[3], Bin Liu[4], Yingbi Zhou[4], Chuan-ming Hao[5], Harvey R. Herschman[6], Jane A. Mitchell[1], Nicholas S. Kirkby[1*]

Affiliations:

1: National Heart & Lung Institute, Imperial College London, London, UK

2: Blizard Institute, Queen Mary University of London, London, UK

3: Clinical Pharmacology and Pharmacotherapy Department, Goethe University, Frankfurt, Germany

4: Cardiovascular Research Centre, Shantou University Medical College, Shantou, China

5: Division of Nephrology, Huashan Hospital, Fudan University, Shanghai, China

6: Department of Molecular and Medical Pharmacology, University of California Los Angeles, USA

#: These authors contributed equally to the study

*: To whom correspondence should be addressed

SUPPLEMENTAL MATERIAL

Supplemental Tables

**mRNA** *(Fold vs. aorta Ptgis)*	**Aorta**	**Renal Cortex**	**Heart**	**Renal Medulla**	**Spleen**	**Colon**	**Lung**
Ptgis	1.00 ± 0.24	0.21 ± 0.08	0.20 ± 0.06	0.26 ± 0.05	0.28 ± 0.11	0.77 ± 0.16	1.39 ± 0.16
Ptgs1	0.03 ± 0.01	0.90 ± 0.45	0.09 ± 0.01	1.03 ± 0.24	1.20 ± 0.48	1.98 ± 0.57	1.00 ± 0.16
Ptgs2	0.004 ± 0.001	0.004 ± 0.001	0.003 ± 0.001	0.049 ± 0.020	0.004 ± 0.001	0.216 ± 0.078	0.069 ± 0.023

Table S1 Expression of prostacyclin synthase and cyclo-oxygenase isoforms in whole mouse tissue Gene expression by RT-qPCR of prostacyclin synthase (*Ptgis*), cyclo-oxygenase-1 (*Ptgs1*) and cyclo-oxygenase-2 (*Ptgs2*) in a selection of mouse tissues. Data are expressed relative to aortic *Ptgis* expression values after normalisation to expression of housekeeping genes (18S and GAPDH). Data are mean ± SEM.

[6kPGF$_{1α}$] (ng/ml)	Heart	Lung	Kidney	Colon	Spleen
**Flox Ctrl**	6.0 ± 2.1	53.5 ± 22.0	10.0 ± 1.8	58.3 ± 19.3	14.7 ± 5.3
**EC/PT COX1 KO**	1.6 ± 0.6	45.8 ± 20.2	15.7 ± 8.4	72.8 ± 23.6	9.9 ± 3.2
**Flox Ctrl**	2.6 ± 0.5	30.1 ± 4.6	15.3 ± 1.6	51.9 ± 7.9	28.7 ± 6.6
**EC/PT COX2 KO**	2.4 ± 0.4	39.1 ± 10.0	16.3 ± 1.8	45.4 ± 7.4	21.7 ± 6.1
**Flox Ctrl**	3.3 ± 1.7	13.6 ± 2.5	3.3 ± 0.6	21.3 ± 3.1	3.9 ± 0.5
**EC/PT PGIS KO**	1.2 ± 0.2	17.3 ± 1.1	3.2 ± 0.4	26.5 ± 4.8	4.1 ± 0.5

Table S2 Effect of endothelial/platelet cyclo-oxygenase-1, cyclo-oxygenase-2 and prostacyclin synthase deletion on tissue prostacyclin release Prostacyclin release (measured as 6kPGF$_{1α}$ after A23187 Ca^{2+} ionophore 30μM stimulation) from isolated aorta, heart, lung, renal medulla, renal cortex, colon and spleen from endothelial/platelet cyclo-oxygenase-1 knockout (EC/PT COX1 KO), endothelial/platelet cyclo-oxygenase-2 knockout (EC/PT COX2 KO) and endothelial/platelet prostacyclin synthase knockout mice (EC/PT PGIS KO) each compared to their own respective floxed littermate control mice (Flox Ctrl). n=4-13. Data are mean ± SEM. All p>0.05 by unpaired t-test.

Expression ($2^{-\Delta Ct}$)	Endothelial cells	Fibroblasts (Adventitial)	Fibroblasts (Peri-bronchial)	Fibroblasts (Alveolar)
Cdh5 (Endothelial marker)	0.131 ± 0.015	0.008 ± 0.003 *	0.001 ± 0.000 *	0.000 ± 0.000 *
Acta2 (Smooth muscle marker)	0.005 ± 0.000	0.007 ± 0.001	0.007 ± 0.001	0.007 ± 0.000
Cspg4 (Pericyte marker)	0.001 ± 0.000	0.000 ± 0.000	0.001 ± 0.000	0.001 ± 0.000
Pdgfrb (Pericyte marker)	0.001 ± 0.000	0.002 ± 0.001	0.001 ± 0.000	0.000 ± 0.000

Table S3 Cell-type marker gene expression in FACS isolated mouse lung cell populations Expression of cell-specific marker genes by RT-qPCR in select populations freshly isolated from wild-type mouse lung by FACS according to the scheme described in Figure 4. n=6. Data are expressed as linearised C_T values after correction for housekeeping gene expression (18S and GAPDH) and displated as mean ± SEM. *, $p<0.05$ versus the endothelial cell fraction by repeated measures one-way ANOVA with Holm-Sidak post-test.

[6kPGF$_{1\alpha}$] (ng/ml)	Aorta	Heart	Lung	Kidney	Colon	Spleen
**Flox Ctrl**	11.5 ± 1.8	3.0 ± 0.6	27.8 ± 4.5	4.6 ± 0.6	18.3 ± 2.4	8.8 ± 2.0
**Fibro COX1 KO**	13.2 ± 3.4	2.9 ± 0.6	14.9 ± 2.6 *	5.4 ± 1.2	12.3 ± 2.0	6.7 ± 2.1

Table S4 Effect of fibroblast cyclo-oxygenase-1 deletion on tissue prostacyclin release Prostacyclin release (measured as 6kPGF$_{1\alpha}$ after A23187 Ca^{2+} ionophore 30μM stimulation) from isolated aorta, heart, lung, kidney, colon and spleen from fibroblast cyclo-oxygenase-1 knockout (Fibro COX1 KO) and floxed littermate control animals (Flox Ctrl). n=9-16. Data are mean ± SEM. *, $p<0.05$ by unpaired t-test.

Supplemental Figures

(A)

Strain	Evidence for deletion in target cells	Evidence for retention in non-target cells
**EC COX1 KO**<br>*(Ptgs1$^{flox/flox}$; VEC-iCre)*	Loss of COX1 protein (immunohistochemistry) in aortic endothelial cells (Mitchell et al. Circ Res. 2019)	Retention of blood TXB_2 formation (widely accepted to indicate platelet COX1 activity) (Mitchell et al. Circ Res. 2019)
**SMC COX1 KO**<br>*(Ptgs1$^{flox/flox}$; Sm22a-Cre)*	Loss of COX1 mRNA (qPCR) in aortic smooth muscle cells (Mitchell et al. Circ Res. 2019)	Retention of COX1 mRNA (qPCR) in aortic endothelial cells (Mitchell et al. Circ Res. 2019)
**EC/PT COX1 KO**<br>*(Ptgs1$^{flox/flox}$; Tie2-Cre)*	Loss of COX1 protein (immunohistochemistry) in aortic endothelial cells and TXB_2 formation by blood (Mitchell et al. Circ Res. 2019).	Retention of COX1 mRNA (qPCR) in whole lung (panel B).
**Fibro COX1 KO**<br>*(Ptgs1$^{flox/flox}$; Fsp1-Cre)*	Loss of COX1 mRNA (qPCR) in fibroblasts from lung explants (panel C) and reduction in whole lung (panel D)	Retention of COX1 mRNA (qPCR) in aorta (panel E)
**EC/PT PGIS KO**<br>*(Ptgis$^{flox/flox}$; Tie2-Cre)*	Loss of COX1 protein (Western blotting) and COX1 mRNA (qPCR) in kidney (Cao et al. Pflugers Arch. 2019).	Not directly assessed. *This can be considered a limitation of our use of this model.*
**EC/PT COX2 KO**<br>*(Ptgs2$^{flox/flox}$; Tie2-Cre)*	Loss of induced COX2 protein (immunohistochemistry) in aortic endothelial cells (Mitchell et al. Circ Res. 2019)	Retention of COX2 mRNA (qPCR) in brain (panel F)

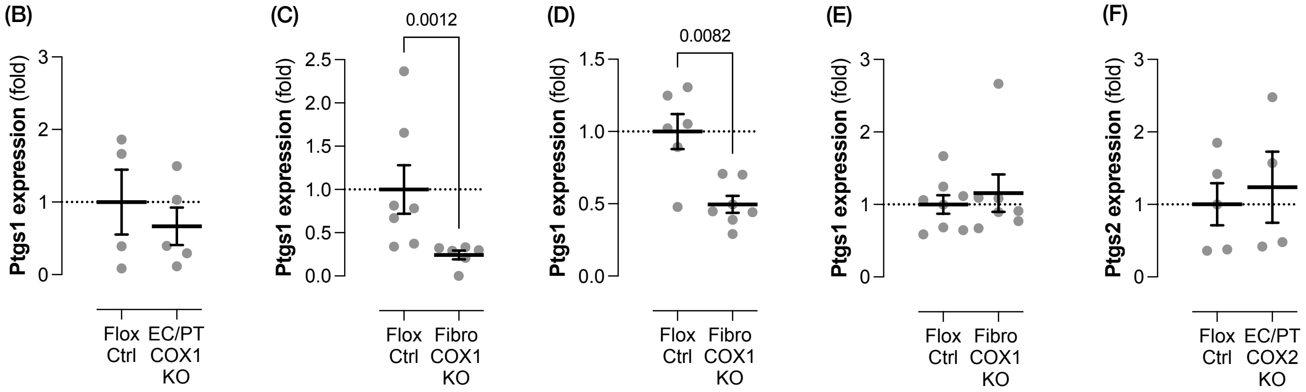

Figure S1 Validation data for selective target gene deletion in cell-specific knockout strains. (A) Summary of validation data (positive and negative) for endothelial (EC), smooth muscle (SMC), endothelial/platelet (EC/PT) and fibroblast (Fibro) cyclo-oxygenase-1 (COX1 KO), cyclo-oxygenase-2 (COX2 KO) or prostacyclin synthase (PGIS KO) knockout mice. Cyclo-oxygenase-1 gene (Ptgs1) expression in (B) whole lung from EC/PT COX1 KO mice and (C) cultured fibroblasts grown from lung explants, (D) whole lung and (E) aorta from Fibro COX1 KO mice. (F) Cyclo-oxygenase-2 gene (Ptgs2) expression in brain from EC/PT COX2 KO mice. Data are mean ± SEM with p values by unpaired t-test (B, D, G) or Mann-Whitney U-test (C, E) indicated where $p<0.05$.

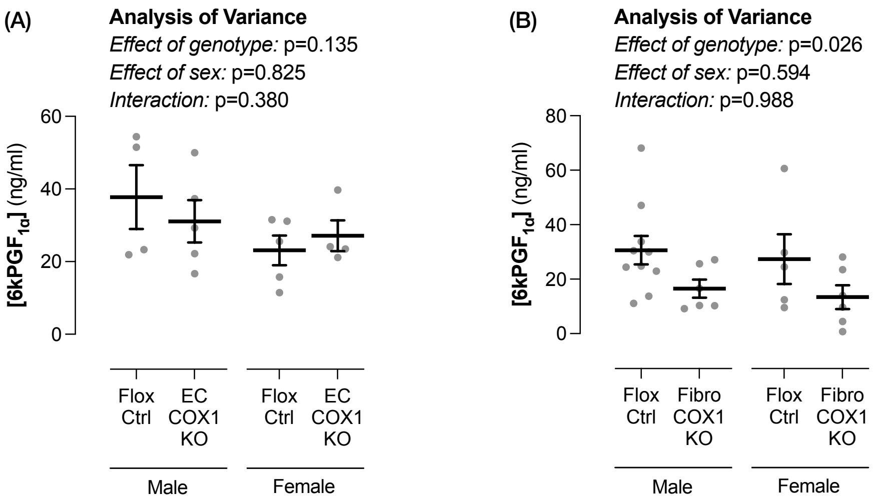

Figure S2 Interaction between sex and ex vivo prostacyclin release from lung of (A) endothelial-specific and (B) fibroblast-specific cyclo-oxygenase-1 knockout mice (A) Prostacyclin release (measured as $6kPGF_{1\alpha}$ after A23187 Ca^{2+} ionophore 30μM stimulation) from lung parenchyma segments from (A) endothelial cyclo-oxygenase-1 knockout (EC COX1 KO) and (B) fibroblast cyclo-oxygenase-1 knockout mice (Fibro COX1 KO; n=5-10) versus their respective floxed littermate control animals (Flox Ctrl; n=4-5), sub-divided by animal sex. Data are mean ± SEM with p values given for the effect of genotype, sex and interaction by two-way ANOVA.

(A)

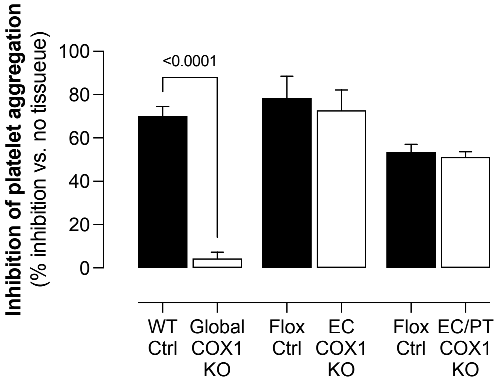

(B)

U46619-induced aggregation (%)	Wild-type vs Global COX-1 KO	Flox Ctrl vs. EC COX1 KO	Flox Ctrl vs. EC/PT COX1 KO
**PRP alone**	60.8 ± 15.4	76.5 ± 1.6	75.1 ± 0.4
**PRP + Lung from Ctrl strain**	22.7 ± 4.9	16.7 ± 7.9	35.0 ± 2.9
**PRP + Lung from KO strain**	56.1 ± 11.8	21.2 ± 7.4	36.6 ± 1.7

Figure S3 Effect of lung from global and endothelial-specific COX-1 knockout models on aggregation of human platelets in vitro Bioassay of prostacyclin activity as inhibition of human platelet aggregation by lung parenchyma from global cyclo-oxygeanse-1 knockout mice (Global COX1 KO) and matched wild-type controls (WT Ctrl) or from endothelial cyclo-oxygenase-1 knockout mice (EC COX1 KO), endothelial/platelet cyclo-oxygenase-1 knockout mice (EC/ PT COX1 KO) and their respective floxed littermate controls (Flox Ctrl) (n=3-5 mice/group). Data are shown as (A) inhibition vs PRP alone and (B) absolute percentage aggregation induced by U46619 under each condition. Data are mean ± SEM with p values by Mann-Whitney U-test indicated where p<0.05.

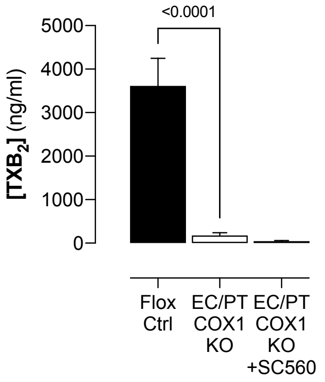

Figure S4 Effect of SC-560 on platelet thromboxane levels in endothelial/platelet cyclo-oxygenase-1 knockout mice Thromboxane release (measured as thromboxane B_2; TXB_2; after A23187 Ca^{2+} ionophore 30μM stimulation) from whole blood from endothelial/platelet cyclo-oxygenase-1 knockout mice (EC/PT COX1 KO) treated with the cyclo-oxygenase-1 inhibitor, SC-560 (10mg/kg; iv, 15 mins) or vehicle (5% DMSO) or vehicle-treated floxed littermate control animals (Flox Ctrl). n=5. Data are mean ± SEM with p values by one-way ANOVA with Holm-Sidak post-test indicated where $p<0.05$.

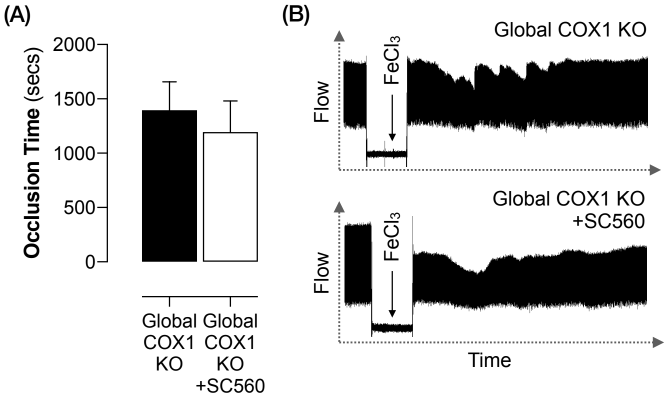

Figure S5 Effect of SC-560 on thrombosis in global cyclo-oxygenase-1 knockout mice Thrombotic occlusion time (A; n=7) and representative blood flow traces (B) after carotid artery $FeCl_3$ injury in vivo in global cyclo-oxygenase-1 knockout mice (Global COX1 KO) treated with SC-560 (10mg/kg; iv, 15 mins) or vehicle (5% DMSO). Data are mean ± SEM with p values by Mann-Whitney U-test indicated where $p<0.05$.

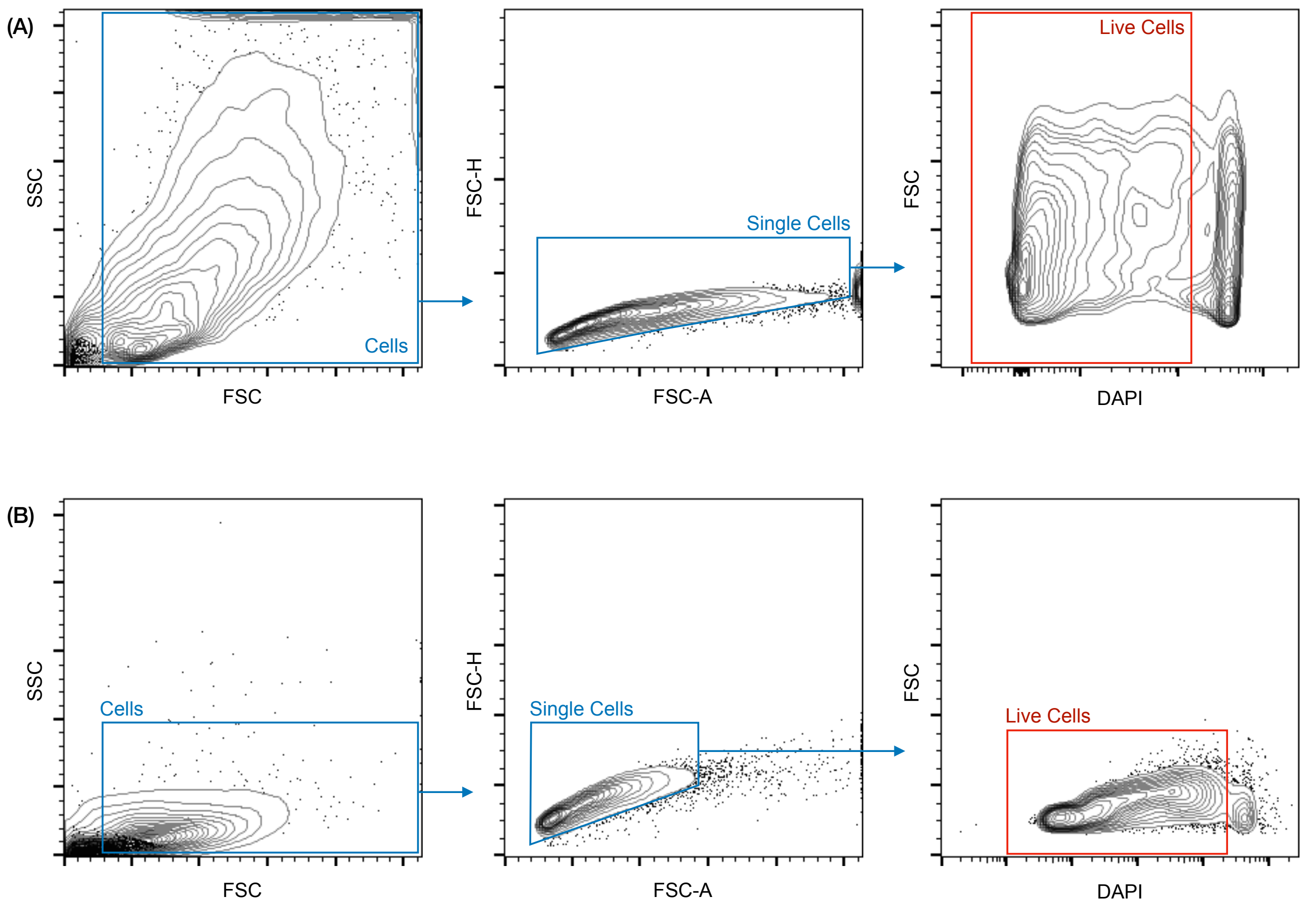

Figure S6 Example plots showing FACS gating for exclusion of debris, doublets and dead cells from (A) mouse and (B) lung cells. Prior to cell type selection based on surface marker expression, forward scatter (FSC) area (FSC-A) and height (FSH-H), side scatter (SSC) and DAPI exclusion properties were used to identify populations of single, live cells from digests of (A) mouse and (B) human lung.

Major Resources Table

Wild-type Animals

Species	Source	Background Strain	Sex	Persistent ID / URL
Mouse	Charles River Labs	C57Bl/6J	M and F	https://www.criver.com/products-services/find-model/c57bl6-mouse

Genetically Modified Animals

Strain	Species	Source	Background Strain	Persistent ID / URL
Ptgs1 -/-	Mouse	*Langenbach et al*	C57Bl/6J	DOI: 10.1016/0092-8674(95)90126-4
Ptgs1 flox	Mouse	Jackson Labs	C57Bl/6J and 129S4/SvJae	https://www.jax.org/strain/030884
Ptgs2 flox	Mouse	Jackson Labs	C57Bl/6J and 129S4/SvJae	https://www.jax.org/strain/030785
Ptgis flox	Mouse	In house	C57Bl/6J	DOI: 10.1007/s00424-018-2229-6
ROSA mT/mG	Mouse	Jackson Labs	C57Bl/6J and 129S4/SvJae	https://www.jax.org/strain/007676
Sm22a Cre	Mouse	Jackson Labs	C57Bl/6J	https://www.jax.org/strain/017491
Tie2 Cre	Mouse	Jackson Labs	C57Bl/6J	https://www.jax.org/strain/008863
Cdh5 iCre	Mouse	Ximbio	C57Bl/6J	https://ximbio.com/reagent/151520/
Fsp1 Cre	Mouse	Jackson Labs	C57Bl/6J and BALB/cByJ	https://www.jax.org/strain/012641

Antibodies

Target antigen	Label	Supplier	Catalog #	Working conc.	Persistent ID / URL
Mouse EpCAM	PE	Biolegend	118205	10µg/ml	https://www.biolegend.com/en-us/products/pe-anti-mouse-cd326-ep-cam-antibody-4726
Mouse CD41	APC/Cy7	Biolegend	133928	10µg/ml	https://www.biolegend.com/en-us/products/apc-cyanine7-anti-mouse-cd41-antibody-13014
Mouse CD41	PE	Biolegend	133905	2.5µg/ml	https://www.biolegend.com/en-us/products/pe-anti-mouse-cd41-antibody-5897
Mouse CD45	PE/Cy7	Biolegend	103114	2.5µg/ml	https://www.biolegend.com/en-us/products/pe-cyanine7-anti-mouse-cd45-antibody-1903
Mouse CD45	PE	Biolegend	103105	2.5µg/ml	https://www.biolegend.com/en-us/products/pe-anti-mouse-cd45-antibody-100
Mouse CD31	PerCP/Cy5.5	Biolegend	102522	2.5µg/ml	https://www.biolegend.com/en-us/products/percp-cyanine5-5-anti-mouse-cd31-antibody-12866
Mouse CD31	AF488	Biolegend	102514	20µg/ml	https://www.biolegend.com/en-us/products/alexa-fluor-488-anti-mouse-cd31-antibody-3093
Mouse Podoplanin	APC	Biolegend	127409	2.5µg/ml	https://www.biolegend.com/en-us/products/apc-anti-mouse-podoplanin-antibody-6656

Mouse Ter119	PE	Biolegend	116207	2.5μg/ml	https://www.biolegend.com/en-us/products/pe-anti-mouse-ter-119-erythroid-cells-antibody-1867
Mouse Sca1	PE/Cy7	Biolegend	108113	2.5μg/ml	https://www.biolegend.com/en-us/products/pe-cyanine7-anti-mouse-ly-6a-e-sca-1-antibody-3137
Mouse PDGFRα	APC	Biolegend	135907	10μg/ml	https://www.biolegend.com/en-us/products/apc-anti-mouse-cd140a-antibody-6439
Mouse CD9	APC/Fire750	Biolegend	124813	5μg/ml	https://www.biolegend.com/en-us/products/apcfire-750-anti-mouse-cd9-antibody-16761
Mouse CD146	AF488	Biolegend	134707	5μg/ml	https://www.biolegend.com/en-us/products/alexa-fluor-488-anti-mouse-cd146-antibody-6864
Human EpCAM	PE	Biolegend	324206	1.25μg/ml	https://www.biolegend.com/en-us/products/pe-anti-human-cd326-epcam-antibody-3757
Human EpCAM	FITC	Biolegend	324203	2.5μg/ml	https://www.biolegend.com/en-us/products/fitc-anti-human-cd326-epcam-antibody-3756
Human CD41	APC/Cy7	Biolegend	303715	5μg/ml	https://www.biolegend.com/en-us/products/apc-cyanine7-anti-human-cd41-antibody-7113
Human CD45	PerCP/Cy5.5	Biolegend	368503	2.5μg/ml	https://www.biolegend.com/en-us/products/percp-cyanine5-5-anti-human-cd45-antibody-12415
Human CD31	FITC	Biolegend	303104	10μg/ml	https://www.biolegend.com/en-us/products/fitc-anti-human-cd31-antibody-881
Human Podoplanin	APC	Biolegend	337022	5μg/ml	https://www.biolegend.com/en-us/products/fitc-anti-human-cd31-antibody-881
Human CD235a	FITC	Biolegend	349103	5μg/ml	https://www.biolegend.com/en-us/products/fitc-anti-human-cd235a-glycophorin-a-antibody-6701
Human CD146	PE	Biolegend	342003	20 μg/ml	https://www.biolegend.com/en-us/products/pe-anti-human-cd146-muc18-mel-cam-antibody-5874

Cultured Cells

**Name**	**Supplier**	**Sex**	**Persistent ID / URL**
Primary human lung microvascular endothelial cells	Promocell	1 female and 2 male donors	https://promocell.com/product/human-pulmonary-microvascular-endothelial-cells-hpmec/
Primary human lung fibroblasts	Lonza	3 female donors	https://promocell.com/product/human-pulmonary-fibroblasts-hpf/

ARRIVE Reporting

Study Design

Studies used both male and female animals and were matched for age and sex according to the criteria below. For studies employing cell-specific knockout mice, floxed, Cre-negative littermates were used as controls. For studies employing germline (cyclo-oxygenase-1) knockout mice, strain-matched non-littermate wild-type mice were used as controls.

Sample Size

Initial studies of lung prostacyclin release in endothelial cyclo-oxygenase-1 knockout mice were powered from a priori sample size calculations which indicated that n=9 would provide 80% power to detect as 33% reduction in prostacyclin release, using variance estimated from our previous studies of aortic prostacyclin release. Subsequent mechanistic investigations were not the subject of a priori sample size calculations.

Inclusion Criteria

Male or female animals, aged 8-10 weeks old of required genotype.

Exclusion Criteria

Between allocation to experimental groups and performing experiments/collection of samples, animals were only excluded where there were signs of ill health or other concerns for animal welfare.

Randomization

Animals were randomly assigned a unique identifier at weaning and prior to genotyping then assigned to experimental groups in sequential order.

Blinding

Wherever possible/practical, individuals performing experiments were aware only of animal identifier numbers during in vivo procedures and any subsequent sample analysis, with the animal genotypes held by a separate individual and unblinded only once data collection and analysis was complete.
